# Smoking Gun or Circumstantial Evidence? Comparison of Statistical Learning Methods using Functional Annotations for Prioritizing Risk Variants

**DOI:** 10.1038/srep13373

**Published:** 2015-08-24

**Authors:** Sarah A. Gagliano, Reena Ravji, Michael R. Barnes, Michael E. Weale, Jo Knight

**Affiliations:** 1 Campbell Family Mental Health Research Institute, Centre for Addiction and Mental Health, Toronto, Ontario, Canada; 2 Institute of Medical Science, University of Toronto, Toronto, Ontario, Canada; 3 William Harvey Research Institute, Barts and The London School of Medicine and Dentistry, Queen Mary University of London, London, UK; 4 Department of Medical & Molecular Genetics, King’s College London, Guy’s Hospital, London, UK; 5 Department of Psychiatry, University of Toronto, Toronto, Ontario, Canada; 6 Biostatistics Division, Dalla Lana School of Public Health, University of Toronto, Toronto, Ontario, Canada

## Abstract

Although technology has triumphed in facilitating routine genome sequencing, new challenges have been created for the data-analyst. Genome-scale surveys of human variation generate volumes of data that far exceed capabilities for laboratory characterization. By incorporating functional annotations as predictors, statistical learning has been widely investigated for prioritizing genetic variants likely to be associated with complex disease. We compared three published prioritization procedures, which use different statistical learning algorithms and different predictors with regard to the quantity, type and coding. We also explored different combinations of algorithm and annotation set. As an application, we tested which methodology performed best for prioritizing variants using data from a large schizophrenia meta-analysis by the Psychiatric Genomics Consortium. Results suggest that all methods have considerable (and similar) predictive accuracies (AUCs 0.64–0.71) in test set data, but there is more variability in the application to the schizophrenia GWAS. In conclusion, a variety of algorithms and annotations seem to have a similar potential to effectively enrich true risk variants in genome-scale datasets, however none offer more than incremental improvement in prediction. We discuss how methods might be evolved for risk variant prediction to address the impending bottleneck of the new generation of genome re-sequencing studies.

Complex diseases are caused by the interplay of many genetic variants and the environment, and represent a considerable health burden. Genome-wide association studies (GWAS) have had success in identifying some genetic risk factors involved in complex diseases such as inflammatory bowel disease^1^ and schizophrenia^2^. Interrogating the entire genome, exome or even selected genes through next generation sequencing technologies have also identified further risk variants^3^,^4^,^5^,^6^. However, more disease-associated variants, hereafter called risk variants or hits, remain to be discovered. Some risk variants are difficult to detect by current techniques due to limited sample sizes and low effect size of the variants. *In silico* methodologies that integrate evidence over multiple data sources have the potential to unearth some of these risk variants in a cost-effective manner. The novel risk variants that are identified will help illuminate the genetic risk factors involved in complex diseases, which in turn could lead to earlier or more accurate diagnoses, and the development of personalized treatment options.

Risk variants show enrichment in functional annotations, such as DNase I hypersensitive sites, transcription factor binding sites, and histone modifications (for example^7^,^8^,^9^). Several groups have gone further with the results of enrichment by incorporating functional annotations as predictor variables in statistical learning frameworks to prioritize genetic variants for further study^10^,^11^,^12^. These statistical learning algorithms use the functional annotations to define a model that provides some measure of whether a variant is likely to increase the risk of manifesting a complex trait. However, understanding the relative merits of these approaches requires a thorough investigation into which statistical learning algorithm and/or which combination of functional annotations most effectively identifies novel risk variants.

There are many aspects to consider in the statistical learning framework (Supplementary Fig. 1). The genetic data input consists of both known risk variants and corresponding control variants (those with no evidence for risk effect); the classifier is used to discriminate between the two. Known risk variants may be identified from sources, such as the National Health Genome Research Institute (NHGRI) GWAS Catalogue^13^, the ClinVar database^14^, and the Human Gene Mutation Database (HGMD)^15^. In addition, the variants can be simulated; for example, Kircher *et al.* used an empirical model of sequence evolution with local adjustment of mutation rates^11^. In this way, the simulated variants would contain *de novo* pathogenic mutations. The goal of these methods is to identify disease-causing variants, but their application can differ depending on whether the data under consideration consist of densely mapped variants, as in sequence data, or coarsely mapped variants, as in GWAS data. The use of different classifiers has the effect of refining the goal, in that coarsely mapped variants may tag other variants in high linkage disequilibrium, and so the functional characteristics of these other variants should be taken into account. The methods we investigate have been applied to both types of data^16^,^17^.

With regard to the functional annotations, some come from experimental procedures while others are predicted computationally. Examples include genomic and epigenomic annotations that can be incorporated from various online browsers and collections such as the Ensembl Variant Effect Predictor (VEP)^18^ and the Encyclopedia of DNA Elements (ENCODE) Project^19^. Whether a variant is assigned the annotations that can be attributed to itself only or to other variants with which it is in linkage disequilibrium can also refine the goal of the method.

Finally, there are numerous statistical learning algorithms from which to choose. These algorithms must be able to handle the features of the functional data: correlations among predictor variables, and a large quantity of both samples and predictor variables.

In this paper, we compared the performance of three published methods that differ in annotation set, algorithm and genetic variants, including the classifier: a regularized regression called elastic net from Gagliano *et al.* (14 annotations)^10^, a modified random forest from Ritchie *et al.* (174 annotations)^12^ called GWAVA and a support vector machine from Kircher *et al.* (949 annotations, expanded from 63 unique annotations) called CADD^11^. These three papers describe algorithms capable of incorporating a large number of genetic variants labeled with multiple functional annotations, and can output a prediction score for each variant; hence, they are highly comparable. Although other methods exist to prioritize genetic risk variants, such as through the use hierarchical Bayesian analysis^20^,^21^, these require genetic association statistics for each variant for prioritization, and thus were beyond the scope of the comparisons in this paper. We investigate nine model types: combinations of the three different statistical learning algorithms and the three different functional annotation sets (summarized in Table 1). All model types were created for different classifications of hits: the NHGRI GWAS Catalogue^13^ and the Human Gene Mutation Database (HGMD)^15^.

Models based on GWAS data can be tested effectively in current data (we apply those models to the schizophrenia GWAS from the Psychiatric Genomics Consortium).

## Results

Our primary analysis used the NHGRI GWAS Catalogue as the classifier. Risk variants/hits were defined as those variants present in the NHGRI GWAS Catalogue (www.genome.gov/gwastudies, downloaded on August 7, 2014)^13^ with a p-value of equal to or less than the accepted threshold for genome-wide significance, 5 × 10^−8^. A subset of non-hits (that are not in high linkage disequilibrium with the hits) was selected from common GWAS arrays for comparability. For the three annotation sets described above, when working with different classifiers some rare annotations have no variability and hence were not used to build the model. In this analysis none of the 14 annotations from Gagliano *et al.* were invariable, three of the 174 annotations from Ritchie *et al.* were invariable, and 509 of the 949 annotations from Kircher *et al.* were invariable. An independent test set was used to determine accuracy of the models for discriminating hits from non-hits based on the predictive score output from each model. These results are presented below.

### Area under the ROC curve

All the models had similar accuracy as demonstrated by the area under the curve (AUC) in the test set data (Table 2). Models using Kircher *et al.*’s annotations produced slightly higher AUCs compared to the other two annotation sets for the elastic net and random forest algorithms. In particular the combination of elastic net and Kircher *et al.*’s annotations was the only model that produced an AUC with confidence intervals that do not overlap with any of the other models.

The AUC results for the training set were also computed to investigate whether the models were over-fit; that is to say, whether the training set AUC is much higher than the test set AUC. We found that for the Ritchie *et al.* and Kircher *et al.* annotation sets, the random forest models with node size equal to one were prone to over-fitting. For instance, for the random forest model based on the Ritchie *et al.* annotations, the test set and training set AUCs were 0.687 and 0.998, respectively (further data available on request). The over-fitting in the random forest models was solved when the minimum node size was set to 10% of the total sample size. Therefore only the random forest models with the minimum node size equal to 10% of the data are presented in Table 2 and discussed further in the results. These results highlight the importance of ensuring that appropriate parameters are chosen for the algorithms.

### Density and distribution of prediction scores

**Figure 1:**
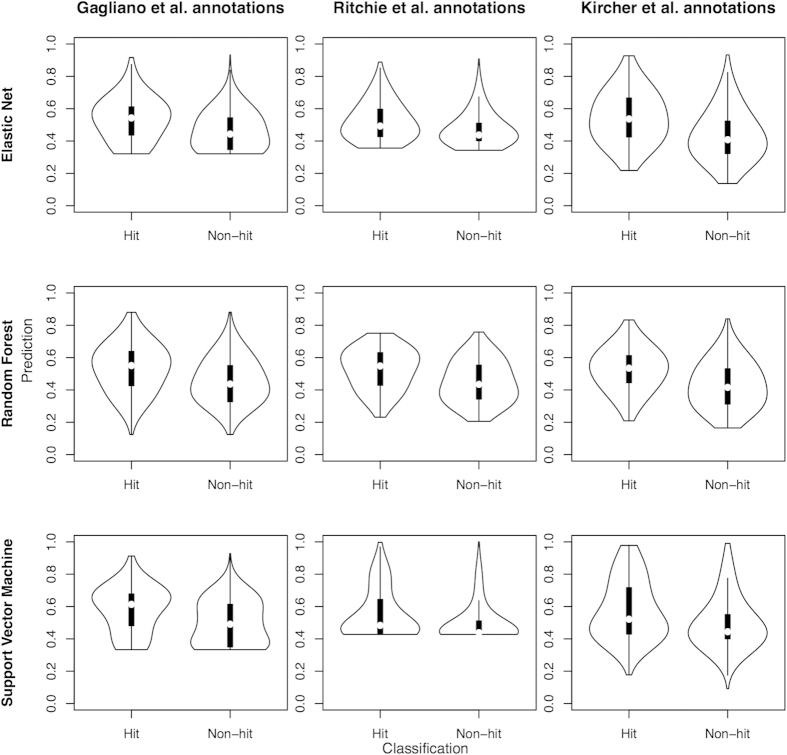
Violin plots showing class separation by prediction scores for the various comparisons using the GWAS Catalogue as the classifier. Hits are variants in the GWAS Catalogue with a genome-wide significant p-value (p ≤ 5 × 10^−8^) and non-hits are those not present in the GWAS Catalogue, but are found on common GWAS arrays for comparison purposes. The non-scaled elastic net models are plotted. The adjusted minimum node size (10%) random forest models are plotted.

Violin plots were constructed by plotting the prediction scores for hits (risk variants) and non-hits separately in order to visualize how well the two classes separated (Fig. 1 and Supplementary Table 1a). The two models with the best AUCs (Kircher *et al.* annotations with elastic net (0.71) and with random forest (0.70)) have comparatively well separated means and relatively normal distributions. In one of the two models with the lowest AUC (Ritchie *et al.* annotations with support vector machine (0.64)), the median prediction score between hits and non-hits is most similar and the distribution is very skewed. Interestingly, one of the mid-range performance models, the Gagliano *et al.* annotations for the support vector machine (0.66) showed evidence of a multimodal distribution where one mode is more common for hits and another for non-hits. However, this effect may simply be due to the comparatively small number of annotations, which lead to a smaller number of possible scores. Generally, the models created using the Kircher *et al.* annotations showed the largest spread of prediction scores for both hits and non-hits. We have also reported the proportion of hits in the top versus the bottom quartiles of the prediction scores in the test set (Supplementary Table 2). In summary the violin plots show that the distributions for hits and non-hits overlapped for all models. However, we see from Supplementary Table 2 that of the variants in the top quartile of prediction scores, there are significantly more hits compared to the lower quartile for all models assessed (p < 2.2 × 10^−16^, chi-square test).

To investigate the consistency of the models we calculated pairwise correlations of the prediction scores in the test set for the various models either holding the algorithm or the annotation set constant. We found that the models with the most correlated scores were those using the Gagliano *et al.* annotation set. Furthermore, the degree of correlation when holding the algorithm constant, but varying the annotation set, was generally not as high as when holding the annotation set constant (Supplementary Table 3).

### Feature selection within elastic net and random forest

More does not necessarily equal better as not all the annotations may be relevant to predicting risk variants. Generally, not all of the functional annotations in the annotation sets were used to create the various models. For instance of the variable features, elastic net assigned non-zero Beta coefficients to 9 out of 14 annotations, 12 out of 171, and 16 out of 432. Random forest assigned non-zero Gini importance values to all of the 14, 131 out of 171, and 239 out of 432. All of these models had similar performance in the test sets (AUCs ranging from 0.68 to 0.70 for the random forest models and 0.65 to 0.71 for the elastic net models). The results suggest that elastic net has a more stringent feature selection implementation than random forest. The support vector machine models always assigned non-zero feature weights, as support vector machine does not intrinsically perform feature selection, as does elastic net and random forest. Thus, we inputted only those annotations with a non-zero Beta coefficient from the elastic net models into the support vector machine models (see **Methods**).

### Importance of the functional annotations

Different combinations of annotations can be used to obtain models with similar predictive accuracy. Furthermore, it is difficult to interpret the importance of the annotations for numerous reasons, some of which are discussed below.

All three annotation sets contained a mixture of binary variables and continuous variables. For Kircher *et al.*’s annotations, background selection (the annotation with the widest continuous scale that ranged from 0 to 1000) came up as most important for predicting the class label in the random forest model. This bias for random forest preferentially selecting annotations measured on a continuous scale has been previously described^22^. When making a decision at a node, continuous annotations can be used multiple times at varying cut-offs to split the data. In this way, functional annotations measured on a continuous scale are incorporated more often into the forest compared to non-continuous annotations, and thus obtain higher variable importance measures^22^,^23^.

It is also difficult to interpret the variable importance measures derived from elastic net because this algorithm is not scale invariant. Using Gagliano *et al.*’s annotations with elastic net, we compared the models created with scaled (all annotations have a standard deviation of 1 and a mean of 0) versus non-scaled annotations. Although the AUCs for both models were nearly identical, the assigned Beta coefficients differed (Supplementary Fig. 2). When we do standardize the scale, we find that the order of importance of coefficients replicates that of the random forest model. However, standardizing a set of largely binary variables removes the effect linked to the frequency, and thus skews the biological representation. So it is not clear that scaling is the best approach.

Although the focus is not about annotations we have provided details of the various importance measures in supplementary data: see Supplementary Tables 9–17 and Supplementary Figures 5–13 for the feature importance measures from all the models based on the GWAS Catalogue as the classifier. In the primary analysis transcription factor binding sites were consistently in the top three annotations for the Gagliano *et al.* annotations for all three algorithms, but there were no other clear patterns with regard to important annotations for the Ritchie *et al.* or Kircher *et al.* annotation sets. In summary, different annotations came up as most important for the various models regardless of predictive accuracy.

### Performance for complex disease variants: Application to Schizophrenia GWAS

**Figure 2:**
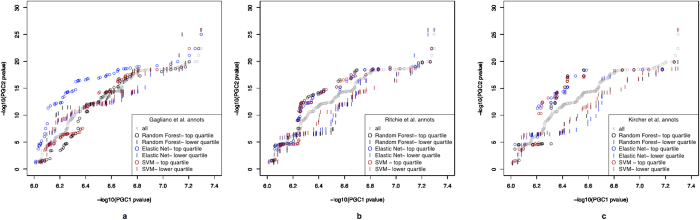
Quantile-quantile plots of PGC1 sub-genome-wide-significant variants (5 × 10^−8^ < p < 1 × 10^−6^) stratified by prediction score for the various models based on the GWAS Catalogue classifier, and plotted by PGC2 p-values. PGC1 p-values are plotted on the x-axis and PGC2 p-values are plotted on the y-axis. Models grouped by annotation set: Gagliano *et al.* (**a**) Ritchie *et al.* (**b**) and Kircher *et al.* annotations (**c**). The lower quartile genetic variants are those PGC1 sub-genome-wide-significant variants that were assigned the lowest prediction scores (in the first quartile), and the top quartile variants are those with the highest prediction scores (in the fourth quartile).

Various quantile-quantile plots were constructed in order to compare which models showed greater separation of the schizophrenia GWAS p-values for high scoring and low scoring functional variants. For all of the models, scores were obtained for the sub-genome-wide-significant variants (5 × 10^−8^ < p < 1 × 10^−6^) from the first round of the GWAS by the Psychiatric Genomics Consortium (PGC1)^24^. The PGC1 p-values were plotted on the x-axis and the p-values from the second larger round of the schizophrenia GWAS (PGC2)^2^ were plotted on the y-axis (Fig. 2). (The results from PGC2 were not used to train the model.) Plots were constructed where annotations were held constant but the algorithm differed. For instance, for the 14 annotations from Gagliano *et al.* we plotted the models from the three algorithms in one plot. Furthermore, models from the same algorithm but varying by annotation set were compared (Supplementary Fig. 3). We have also reported the proportion of hits in the top versus the bottom quartiles of the prediction scores in the test set (Supplementary Table 4). With regard to the functional annotation set, the separation of the novel associated variants from the non-associated in the sub-genome-wide-significant variants was best exhibited in the quantile-quantile plots when using either the Kircher *et al.* or Ritchie *et al.* annotation sets. Regardless of annotation set, the elastic net models consistently showed good separation. For all algorithms using either the Ritchie *et al.* or Kircher *et al.* annotations, the PGC1 sub-genome-wide-significant variants that have the highest prediction scores (within the top quartile) consistently contain a higher proportion of GWAS significant variants from the second round of the schizophrenia GWAS (p ≤ 5 × 10^−8^) compared to the variants that have scores in the lower quartile. The elastic net models too, regardless of annotation set, showed this pattern. Although these patterns are not all statistically significant, it is notable that the biggest positive difference comes from using the Ritchie *et al.* annotations with the elastic net algorithm, and the most significant difference between the proportion of GWAS significant variants in the top quartile compared to the proportion in the lower quartile comes from the Kircher *et al.* annotations using the elastic net algorithm; (there are more variants available in the Kircher *et al.* model than the Ritchie *et al.* model). The Gagliano *et al.* annotations performed very poorly with both the random forest and support vector machine algorithms since the variants with low prediction scores were more likely to be hits than those with high scores. This is a result of the PGC2 hits not being enriched in two of the top annotations for the Gagliano *et al.* models using either the random forest or support vector machine algorithms, H3K4Me3 and H3K27Ac. In the GWAS Catalogue analysis of the variants that possess the H3K4Me3 and H3K27Ac marks, nearly 70% are hits and the remainder are non-hits. In comparison, of the PGC1 sub-genome-wide-threshold variants that possess those two annotations, only 21% are PGC2 hits, and the remaining variants are non-hits.

The results for the application to the schizophrenia GWAS did not always reflect the AUCs from the training data. For instance, a poor performing model in terms of AUC based on the test set, elastic net with the Ritchie *et al.* annotations, performed well in the GWAS application. All in all, the accuracy of the resulting models should be assessed by various means, including (but not limited to) theoretical models such as the ROC curve, as well as empirical approaches such as applying the model using data from one study and evaluating its performance on independent data with gold standard answers.

### HGMD Analysis

In an attempt to apply the algorithms and annotation set combinations to whole genome sequencing data, and indeed fine-mapping studies, rather than just GWAS, a different classifier was used to identify hits and non-hits, the Human Gene Mutation Database (HGMD). We conducted two analyses with subsets of the public release of HGMD. In the first, we took all the variants (single nucleotide polymorphisms) in HGMD and chose controls that fell within a kilobase of either side from the HGMD variant. In this analysis one of the 14 annotations from Gagliano *et al.* was invariable, eight of the 174 annotations from Ritchie *et al.* were invariable, and 396 of the 949 annotations from Kircher *et al.* were invariable. Secondly, models based on the subset of non-exonic HGMD variants and non-exonic control variants were assessed. This second set of models was created in an effort to overcome the ascertainment bias inherent in HGMD related to genes. In this analysis two of the 14 annotations from Gagliano *et al.* were invariable, 16 of the 174 annotations from Ritchie *et al.* were invariable, and 756 of the 949 annotations from Kircher *et al.* were invariable.

The models for the analysis using all of the HGMD variants using either the Ritchie *et al.* or Kircher *et al.* annotations had high predictive accuracy (Table 3). The AUCs for the non-exonic HGMD analysis were more comparable to the ones obtained for the primary analysis using the GWAS Catalogue as the classifier (Table 4), but again the annotations from Ritchie *et al.* and Kircher *et al.* performed better.

Similar to the analysis using the GWAS Catalogue as the classifier, for the HGMD analysis models the features that came up as most important tended to vary depending on the algorithm and are difficult to interpret. It is however notable that genic annotations featured highly (see Supplementary Tables 18–26 and Supplementary Figures 14–22). For the Gagliano *et al.* annotations, the top annotation (or the second most important in the case of support vector machine) was nonsynonymous SNPs. For the Kircher *et al.* annotations, the top annotations for the random forest and support vector machine models were related to the coding sequence or nonsynonymous SNPs. The top annotation for elastic net was CpG. For the Ritchie *et al.* annotations, the top two annotations were coding sequence and exon for both the random forest and support vector machine models. For elastic net, the top two annotations were donor and coding sequence. The importance of genic features is likely linked to bias in the data, which will be examined further in the **Discussion**.

The HGMD analysis in which only non-exonic HGMD and control variants were considered seemed to overcome this bias towards genes or positions relative to genes. Interestingly, for all algorithms, the top annotation for the Gagliano *et al.* annotation set was DNase I hypersensitive sites, but we caution against making biological inferences on the top annotations for the reasons outlined above (see Supplementary Tables 27–35 and Supplementary Figures 23–31).

## Discussion

We found that the three algorithms assessed here, elastic net, random forest and the linear support vector machine show comparable accuracy in the GWAS test data. The Kircher *et al.* annotations trained using the elastic net algorithm have the highest AUC. When applied to real data, several models show the potential to prioritize novel hits, with the exception of the random forest and support vector machine models using the Gagliano *et al.* annotations. However, this was just one real dataset and further studies would need to be assessed to validate this conclusion. Under the conditions employed in our analysis, none of the models were over-fitted, as demonstrated by verifying that the training set AUC is similar in magnitude to that of the test set.

Furthermore, our results show that various combinations of annotations can create models with similar predictive ability when it comes to identifying risk variants from non-risk variants. One must be wary of making strong conclusions about the relevance of the annotations because of the difficulty in interpretation. The coefficients or variable importance measures are differentially affected by issues such as correlation between the attributes, and whether variables are normalized (for elastic net and support vector machine). This observation makes it difficult to differentiate the predictive power of the functional annotation sets used by each study, at least in the case of GWAS risk variants.

As mentioned in the **Introduction**, the main goals of these methods are to identify those variants that are important for disease risk, which can be applied to identifying novel loci or for fine-mapping at previously implicated loci. The HGMD is designed to contain disease variants, whereas the GWAS Catalogue contains variants associated with disease, but those variants may only be tagging the “causal” variant. GWAS are undertaken to identify the loci containing the variant and may identify the actual causal variant but will more often identify variant in high linkage disequilibrium with the causal variant. Thus, the primary analyses in this paper (using the GWAS Catalogue) may be considered to be about identifying novel loci rather than fine-mapping, and the HGMD analyses may be considered to be more about fine-mapping a specific locus. Furthermore, the Gagliano *et al.* method may be considered to be better suited to identifying novel loci (rather than fine-mapping) because it annotates variants on whether or not the variant itself falls into the base pair range for the functional annotation, but also if that variant has is in linkage disequilibrium (r^2^ > 0.8) with a variant that falls into the range. The Ritchie *et al.* and Kircher *et al.* methods annotate the variants just based on whether the variant itself falls into the base sequence for the functional annotation, and do not look at their linkage disequilibrium proxies. That being said, we also performed the analyses for the Gagliano *et al.* annotations only considering whether the variant itself falls into the sequence for the functional annotation as an additional analysis. The resulting models had very similar accuracy to those models created when the linkage disequilibrium proxies were taken into account (data available on request).

To apply the methods in next generation sequencing data and fine-mapping studies we would ideally use risk variants identified from such studies. Unfortunately, there are not a sufficient number available. We used the HGMD to attempt to extrapolate our findings. However, we believe the high accuracies achieved for the all HGMD models (ie. not the models looking just at non-exonic variants) are driven by the inherent bias of the HGMD data, in that it is largely focused on genes. For the models using only non-exonic HGMD and control variants, the AUCs were considerably lower, with the Kircher *et al.* and Ritchie *et al.* annotation sets clearly out-performing the annotations used by Gagliano *et al.* Yet, this subset of HGMD is a highly derived and filtered set of variants, emphasizing the need for empirical data. The simulation employed by Kircher *et al.* to consider all variants, in which the functional annotations were used to differentiate between millions of high frequency human-derived alleles from the same number of simulated alleles^11^, showed considerable accuracy; further adaptions to this strategy may prove useful.

Compared to the corresponding elastic net or random forest models, the support vector machine models consistently produced slightly lower AUCs for the GWAS Catalogue and all HGMD analyses. This poorer performance may be attributed to the fact that we implemented the most basic kernel type for the support vector machine, a linear kernel. This kernel was chosen in an effort to be consistent with the type of kernel that was utilized by Kircher *et al.*, and with the advantage that computational time remains comparable with the other algorithms (Supplementary Text and Supplementary Tables 4–7). However, a linear kernel may not be best to separate the data. Furthermore, as support vector machine does not intrinsically perform feature selection, we selected a subset of features with a non-zero Beta coefficient from the corresponding analysis using the elastic net algorithm. Use of another method of feature selection may have yielded different results. Our results do not necessarily suggest that the elastic net and random forest algorithms out-perform the support vector machine algorithm, since altering either the kernel type or the functional annotations in the support vector machine models may produce results comparable to the other two algorithms.

There are limitations to this comparison. For example, other statistical learning algorithms, such as a deep neural network^25^, and other annotation sets could be explored. Annotation sets could be phenotype specific, as there is evidence that the level of enrichment of functional information can differ depending on the subset of risk variants selected^26^. For instance, enrichment of disease-specific variants in the GWAS Catalogue can differ in certain cell types, for example for DNase I hypersensitive sites^8^.

Identifying which algorithm and/or annotations identify risk variants with the highest accuracy will help researchers develop a better understanding of the genetic factors involved in complex disease in a cost-effective manner making use of a rich set of publically available functional data. This work helps illuminate the genetic factors involved in disease by making use of existing functional data *in silico*. Increasing knowledge on the etiology of complex disease will allow for earlier or better diagnoses, and the development of personalized treatment and novel therapies.

## Methods

We explored the utility of each of the three algorithms with each of the three functional annotation sets in order to attribute performance differences to the algorithm and/or annotations. A total of nine model types were created.

In the primary analysis, the set of risk variants used for training all the models were based on whether or not a genetic variant is a hit or a non-hit from a genome-wide association study (GWAS). Hits were defined as those variants present in the NHGRI GWAS Catalogue (www.genome.gov/gwastudies, downloaded on August 7, 2014)^13^ with a p-value of equal to or less than 5 × 10^−8^. There were 3,618 unique genetic variants that met these criteria. (Note that at the time of download the novel hits from the second phase of the schizophrenia GWAS from the Psychiatric Genomics Consortium (PGC2)^2^ had not yet been included.) A subset of non-hits was selected from common GWAS arrays (Affymetrix Genome-Wide Human SNP Array 6.0, the Illumina Human1M-Duo Genotyping BeadChip, and the Illumina HumanOmni1-Quad BeadChip). Those non-hits in high linkage disequilibrium (r^2^ > 0.8) with hits were removed from the analyses.

### Functional annotation sets

The data was then annotated using three distinct protocols outlined in each of the three respective papers. The variants were marked with the Gagliano *et al.* annotations available on the website (http://www.camh.ca/en/research/research_areas/genetics_and_epigenetics/Pages/Statistical-Genetics.aspx). Fourteen functional annotations were used by Gagliano *et al.*, two of which were on a continuous scale (two conservation measures, PhyloP and PhastCons), and the remaining were binary, signifying the presence or absence. The binary annotations included those related to genomic context such as the presence in a gene, a splice site or a transcription start site, as well as those from the ENCODE Project^19^ such as three types of histone modifications and DNase I hypersensitivity. For the ENCODE data, functional annotations present in multiple cell lines were grouped together, and genetic variants were annotated accordingly in a binary, present or absent, fashion. Variants were marked with an annotation if they or their linkage disequilibrium proxies fall into the base pair range of the annotation.

To annotate the variants using Ritchie *et al.*’s annotations, the data were entered into the online GWAVA webserver (https://www.sanger.ac.uk/resources/software/gwava/). Ritchie *et al.* investigated 174 functional annotations, some binary and others continuous. They also used ENCODE Project tracks including those investigated in Gagliano *et al.* but not necessarily coded as presence or absence. For instance, for transcription factor binding sites, the number of cell types in which the site was present was used as the annotation. Additionally, variation such as mean heterozygosity and genic and sequence contexts were included. Variants were marked with an annotation if they fall into the base pair range of the annotation.

To obtain Kircher *et al.*’s annotations, the data were entered into the online CADD webserver (http://cadd.gs.washington.edu). However, Kircher *et al.* also imputed missing values, expanded categorical variables, added indicator variables, and included interaction terms. Martin Kircher provided scripts to run on the webserver output to prepare our dataset in accordance with the complete protocol. Kircher *et al.* looked at 63 unique functional annotations, which totaled to 949 once the categorical variables were expanded, and the indicator variables and interaction terms were included. A mixture of continuous, categorical, and binary functional annotations was included. Similar annotations to those used by Gagliano *et al.* and/or Ritchie *et al.* were included, such as ENCODE Project annotations and genic context. Additionally, data from online variant prediction programs (e.g. Sift^27^ and PolyPhen^28^) were incorporated. Variants were marked with an annotation if they fall into the base pair range of the annotation.

### Statistical learning algorithms

The variants were randomly divided; 60% was used for training the models, and the remaining 40% was reserved for testing. Elastic net is a regularized logistic regression, and those models were constructed using the glmnet package in R^29^. A weighting procedure was included to up-weight hits, as described in Knight *et al.*^30^; in brief, the weighting has the effect of equalizing the number of hits and non-hits in the training set. Optimal parameters of lambda and alpha were selected for each elastic net model using 10-fold cross validation. Lambda is an overall penalty parameter. Alpha controls the proportion of weight assigned to both the sum of the absolute value of the coefficients and the sum of the squared value of the coefficients, which affects the degree of their sparsity. A range of combinations of lambda and alpha were investigated. The lambda and corresponding alpha that give a model a deviance one standard deviation above the model with the lowest deviance was selected.

Random forest is a collection of decision trees. The random forest models were implemented in Python using the scikit-learn package^31^. Two sets of random forest models were created, both using 10-fold cross validation. For the first set, we replicated Ritchie *et al.*’s random forest implementation by using scripts (e.g. gwava.py) provided on their online GWAVA FTP site (ftp://ftp.sanger.ac.uk/pub/resources/software/gwava/). For instance, bootstrap sampling was employed to form decision trees from bootstrap subset samples. To address the class imbalance in the datasets, non-hits were down-weighted through the balance_classes function created by Ritchie *et al.* and included in their random forest implementation. The balance_classes function selects a subset of non-hits that is equal to the number of hits in order to grow a tree. Furthermore, the subset of annotations used to determine the node split was set to the square root of the total number of annotations. This setting is the default setting for classification problems to determine the best split at each node of the decision tree^32^. Additionally, as done by Ritchie *et al.*, we used 100 decision trees since we determined that the prediction scores and variable importance measures did not significantly differ past 100 trees.

Ritchie *et al.* used a minimum node size (min_samples_split) of 1. The minimum node size is the minimum number of samples required to split an internal node. We created another set of random forest models in which we adjusted the minimum node size. This parameter is dataset specific, and a recommended setting is 10% of the total dataset^32^. Consider n to be the number of hits in the training dataset. For the second set of random forest models, we set the minimum node size to approximately 10% of 2n.

Support vector machine creates a hyperplane within a decision boundary space defined by support vectors to separate the classes in multidimensional space. The support vector machine models were implemented in Python through the scikit-learn package^31^. Kircher *et al.* did not use a weighting procedure as their training set was already balanced. To compare protocols in an unbiased manner, we used a subset of the training set in which we chose all hits, and randomly selected an equal amount of non-hits. We performed a grid search using the tune function in order to determine the optimal cost parameter for a linear kernel. The cost parameter is a penalty (see chapter 9 in James *et al.*^33^ for details). Feature selection is critical to improving model performance and is intrinsically incorporated by the elastic net and random forest algorithms^34^. Feature selection must be implemented before using support vector machine, as there is no feature selection protocol built in. Kircher *et al.* utilized univariate logistic regression among other methods to select features that best predict genetic risk variants. In this paper our support vector machine models included those annotations that had a non-zero Beta coefficient from the corresponding elastic net models. We chose the annotations found to be important from elastic net, since this algorithm implements a more stringent feature selection protocol compared to random forest (see **Results**).

### Assessment of model performance

We assessed model performance in the test set data by calculating the area under the receiver operating characteristic (ROC) curve using the R package ROCR^35^ (and verified using the R package pROC^36^). 95% confidence intervals were generated using 2000 bootstrap replicates also using pROC^36^. As another measure of model performance, we also examined the distribution of prediction scores assigned to the test set data with the aid of violin plots.

We investigated importance of the functional annotations through the Beta coefficient for elastic net. Similar to the output from a simple logistic regression, the larger coefficients are interpreted as more important to predicting genetic risk variants. For random forest we used Gini importance, which was also used in Ritchie *et al.* Gini importance is a scaled measure of Gini impurity averaged over all trees; it represents the improved capacity for correctly predicting variants that can be directly attributed to the annotation^37^. For support vector machine, feature weights can be obtained related to the construction of the hyperplane when a linear kernel is used^38^.

### Performance for complex disease variants: Application to Schizophrenia GWAS

We tested the performance of the nine models based on the GWAS classifier in a schizophrenia GWAS context. We selected all sub-genome-wide-significant variants (5 × 10^−8^ < p < 1 × 10^−6^) from the first round of the GWAS by the Psychiatric Genomics Consortium (PGC1)^24^. For each of the nine models we obtained prediction scores for these variants and selected the variants from the first and fourth prediction score quartiles. For these variants we extracted the p-values from the larger second round of the GWAS (PGC2)^2^ and plotted these in quantile-quantile plots. Note that there is sample overlap in the discovery cohort (about 30%) of the smaller PGC1 in the larger PGC2. Sample details are provided as a Supplementary Table in the PGC2 paper^2^. We were able to determine for all models whether variants assigned higher scores were enriched in the variants with more significant p-values compared to variants with less significant p-values.

### HGMD analysis

The nine models created by combinations of annotation sets and algorithms were assessed using two sets of the public release of the Human Gene Mutation Database (HGMD) variants provided to Ensembl in the fourth quarter of 2013 (provided by Graham Ritchie). In the first, we took all the variants (single nucleotide polymorphisms) in HGMD (N = 3,391) and chose non-hits/controls that fell within a kilobase of either side from the HGMD variant (for consistency with the way the controls were selected in Ritchie *et al.*^12^). Secondly, models based on the subset of non-exonic HGMD variants (N = 689) and non-exonic control variants were assessed. Additionally, the data was randomly split into 60% for training and 40% for testing. The same procedures for elastic net, random forest and support vector machine used in the GWAS Catalogue analysis were also conducted for the HGMD analyses.

### Comparison of scores from the three papers: Application to Schizophrenia GWAS

In the effort for a more general comparison of the published methods as is, rather than looking specifically at the algorithm and annotations as done above, we additionally conducted the schizophrenia GWAS application using scores for the variants obtained directly from the published papers. This analysis is further described in Supplementary Text and the results are depicted in Supplementary Figure 4 and Supplementary Table 8.Computations were performed on either the CAMH Specialized Computing Cluster (SCC) or the General Purpose Cluster (GPC) supercomputer at the SciNet HPC Consortium [Ref. 39].

## Additional Information

**How to cite this article**: Gagliano, S. A. *et al.* Smoking Gun or Circumstantial Evidence? Comparison of Statistical Learning Methods using Functional Annotations for Prioritizing Risk Variants. *Sci. Rep.* **5**, 13373; doi: 10.1038/srep13373 (2015).

## Supplementary Material

Supplementary Information

## Figures and Tables

**Table 1:** Comparison of the three papers.

	Gagliano *et al.* (PLoS ONE 2014)	Ritchie *et al.* (Nat Methods 2014) “GWAVA”	Kircher *et al.* (Nat Genetics 2014) “CADD”
Functional annotations	n = 14 (ENCODE, eQTLs, PhastCons, Genic context…)	n = 174 (ENCODE, GERP, Genic context…)	n = 63 (expanded to 949) (Ensembl VEP, ENCODE, PolyPhen…)
Risk variants (“Hits”)	NHGRI GWAS Catalogue (p-value ≤ 5 × 10^−8^)	HGMD—“regulatory”	Simulated mutations under neutral model—“gap” sites
Non-risk variants (“Non-hits”)	union of common Illumina and Affymetrix GWAS panels	other variants in 1000 Genomes Project (for example, within 1kb of each HGMD variant)	high-frequency derived human alleles from 1000 Genomes
Classifier algorithm	Elastic net	Random forest	Support vector machine
Training protocol	60% training. 40% reserved for testing	100% training	99% training. 1% reserved for testing

**Table 2:** The area under the curve (AUC) for the GWAS Catalogue comparisons, holding data and classifier constant, while varying algorithm and annotations.

Annotations →	Gagliano *et al*.	Ritchie *et al*.	Kircher *et al*.
Elastic Net	0.67 [0.65–0.68] (0.67)	0.65 [0.63–0.66] (0.67)	0.71 [0.69–0.73] (0.74)
Random Forest (altered minimum node size)	0.67 [0.65–0.68] (0.69)	0.68 [0.66–0.69] (0.72)	0.70 [0.68–0.72] (0.79)
Support Vector Machine (with prior feature selection)	0.66 [0.65–0.68] (0.66)	0.64 [0.63–0.66] (0.66)	0.64 [0.61–0.66] (0.68)

The 95% confidence interval based on 2000 bootstrap replicates (generated using the R package pROC) is shown in square brackets. The AUC in the training set is in parentheses.

**Table 3:** The area under the curve (AUC) for the HGMD comparisons, holding data and classifier constant, while varying algorithm and annotations.

Annotations →	Gagliano *et al*.	Ritchie *et al*.	Kircher *et al*.
Elastic Net	0.66 [0.64–0.67] (0.65)	0.87 [0.86–0.88] (0.88)	0.88 [0.87–0.89] (0.88)
Random Forest (altered minimum node size)	0.65 [0.64–0.66] (0.66)	0.91 [0.90–0.92] (0.91)	0.87 [0.86–0.88] (0.89)
Support Vector Machine (with prior feature selection)	0.63 [0.62–0.64] (0.66)	0.85 [0.83–0.86] (0.86)	0.85 [0.84–0.86] (0.87)

The 95% confidence interval based on 2000 bootstrap replicates (generated using the R package pROC) is shown in square brackets. The AUC in the training set is in parentheses.

**Table 4:** The area under the curve (AUC) for the non-exonic HGMD comparisons, holding data and classifier constant, while varying algorithm and annotations.

Annotations →	Gagliano *et al*.	Ritchie *et al*.	Kircher *et al*.
Elastic Net	0.65 [0.61–0.68] (0.66)	0.77 [0.74–0.80] (0.78)	0.79 [0.76–0.81] (0.80)
Random Forest (altered minimum node size)	0.65 [0.61–0.68] (0.65)	0.80 [0.77–0.82] (0.86)	0.78 [0.75–0.80] (0.85)
Support Vector Machine (with prior feature selection)	0.61 [0.58–0.65] (0.68)	0.68 [0.65–0.72] (0.78)	0.76 [0.73–0.78] (0.82)

The 95% confidence interval based on 2000 bootstrap replicates (generated using the R package pROC) is shown in square brackets. The AUC in the training set is in parentheses.

## References

[b1] Jostins L. *et al.* Host-microbe interactions have shaped the genetic architecture of inflammatory bowel disease. Nature 491, 119–124 (2012). 23128233 10.1038/nature11582 PMC3491803

[b2] Schizophrenia Working Group of the Psychiatric Genomics Consortium. Biological insights from 108 schizophrenia-associated genetic loci. Nature 511, 421–427 (2014). 25056061 10.1038/nature13595 PMC4112379

[b3] Rivas M. A. *et al.* Deep resequencing of GWAS loci identifies independent rare variants associated with inflammatory bowel disease. Nat. Genet. 43, 1066–1073 (2011). 21983784 10.1038/ng.952 PMC3378381

[b4] Epi4K Consortium *et al.* De novo mutations in epileptic encephalopathies. Nature 501, 217–221 (2013). 23934111 10.1038/nature12439 PMC3773011

[b5] Neale B. M. *et al.* Patterns and rates of exonic de novo mutations in autism spectrum disorders. Nature 485, 242–245 (2012). 22495311 10.1038/nature11011 PMC3613847

[b6] De Rubeis S. *et al.* Synaptic, transcriptional and chromatin genes disrupted in autism. Nature 515, 209–215 (2014). 25363760 10.1038/nature13772 PMC4402723

[b7] Disanto G. *et al.* DNase hypersensitive sites and association with multiple sclerosis. Hum Mol Genet 23, 942–8 (2014). 24092328 10.1093/hmg/ddt489

[b8] Maurano M. T. *et al.* Systematic Localization of Common Disease-Associated Variation in Regulatory DNA. Science 337, 1190–1195 (2012). 22955828 10.1126/science.1222794 PMC3771521

[b9] Schaub M. A., Boyle A. P., Kundaje A., Batzoglou S. & Snyder M. Linking disease associations with regulatory information in the human genome. Genome Res 22, 1748–59 (2012). 22955986 10.1101/gr.136127.111 PMC3431491

[b10] Gagliano S. A., Barnes M. R., Weale M. E. & Knight J. A Bayesian method to incorporate hundreds of functional characteristics with association evidence to improve variant prioritization. PLoS ONE 9, e98122 (2014). 24844982 10.1371/journal.pone.0098122 PMC4028284

[b11] Kircher M. *et al.* A general framework for estimating the relative pathogenicity of human genetic variants. Nat. Genet. 46, 310–315 (2014). 24487276 10.1038/ng.2892 PMC3992975

[b12] Ritchie G. R. S., Dunham I., Zeggini E. & Flicek P. Functional annotation of noncoding sequence variants. Nat. Methods 11, 294–296 (2014). 24487584 10.1038/nmeth.2832 PMC5015703

[b13] Hindorff L. A., J. H., Hall P. M., Mehta J. P. & Manolio T. A. A catalog of published genome-wide association studies. (2010). Available at www.genome.gov/gwastudies. Accessed: August 7, 2014.

[b14] Landrum M. J. *et al.* ClinVar: public archive of relationships among sequence variation and human phenotype. Nucleic Acids Res 42, D980–5 (2014). 24234437 10.1093/nar/gkt1113 PMC3965032

[b15] Stenson P. D. *et al.* The Human Gene Mutation Database: 2008 update. Genome Med. 1, 13 (2009). 19348700 10.1186/gm13 PMC2651586

[b16] Parra E., Eaton, K., Kavanagh, P., Edwards, M. & Krithika, S. Association study confirms that two OCA2 polymorphisms are involved in normal skin pigmentation variation in East Asian populations; (Abstract #1963S). Presented at the 64th Annual Meeting of The American Society of Human Genetics (October 19, 2014 in San Diego, CA).

[b17] Griswold, A. J. *et al.* Computational evaluation of the pathogenicity of noncoding sequence variants in autism spectrum disorder; (Abstract #1376T). Presented at the 64th Annual Meeting of The American Society of Human Genetics (October 21, 2014 in San Diego, CA).

[b18] McLaren W. *et al.* Deriving the consequences of genomic variants with the Ensembl API and SNP Effect Predictor. Bioinforma. Oxf. Engl. 26, 2069–2070 (2010). 10.1093/bioinformatics/btq330 PMC2916720 20562413

[b19] The ENCODE Project Consortium. A User’s Guide to the Encyclopedia of DNA Elements (ENCODE). PLoS Biol 9, e1001046 (2011). 21526222 10.1371/journal.pbio.1001046 PMC3079585

[b20] Pickrell J. K. Joint Analysis of Functional Genomic Data and Genome-wide Association Studies of 18 Human Traits. Am J Hum Genet 94, 559–73 (2014). 24702953 10.1016/j.ajhg.2014.03.004 PMC3980523

[b21] Kichaev G. *et al.* Integrating Functional Data to Prioritize Causal Variants in Statistical Fine-Mapping Studies. PLoS Genet. 10, (2014). 10.1371/journal.pgen.1004722 PMC4214605 25357204

[b22] Strobl C., Boulesteix A. L., Zeileis A. & Hothorn T. Bias in random forest variable importance measures: illustrations, sources and a solution. BMC Bioinformatics 8, 25 (2007). 17254353 10.1186/1471-2105-8-25 PMC1796903

[b23] Boulesteix A.-L. Janitza S. Hapfelmeier A. Van Steen, K. & Strobl C. Letter to the Editor: On the term ‘interaction’ and related phrases in the literature on Random Forests. Brief. Bioinform. 16(2), 338–45 (2014). 24723569 10.1093/bib/bbu012 PMC4364067

[b24] Schizophrenia Psychiatric Genome-Wide Association Study (GWAS) Consortium. Genome-wide association study identifies five new schizophrenia loci. Nat. Genet. 43, 969–976 (2011). 21926974 10.1038/ng.940 PMC3303194

[b25] Quang D., Chen Y. & Xie X. DANN: a deep learning approach for annotating the pathogenicity of genetic variants. Bioinforma. Oxf. Engl. 31, 761–763 (2015). 10.1093/bioinformatics/btu703 PMC4341060 25338716

[b26] Farh K. K.-H. *et al.* Genetic and epigenetic fine mapping of causal autoimmune disease variants. Nature 518, 337–343 (2015). 25363779 10.1038/nature13835 PMC4336207

[b27] Ng P. C. & Henikoff S. SIFT: Predicting amino acid changes that affect protein function. Nucleic Acids Res. 31, 3812–3814 (2003). 12824425 10.1093/nar/gkg509 PMC168916

[b28] Adzhubei I.A. *et al.* A method and server for predicting damaging missense mutations. Nat Methods 7, 248–249 (2010). 20354512 10.1038/nmeth0410-248 PMC2855889

[b29] Core Team. R R: A language and environment for statistical computing. R Foundation for Statistical Computing, Vienna, Austria (2013).

[b30] Knight J., Barnes M. R., Breen G. & Weale M. E. Using Functional Annotation for the Empirical Determination of Bayes Factors for Genome-Wide Association Study Analysis. PLoS ONE 6, e14808 (2011). 21556132 10.1371/journal.pone.0014808 PMC3083387

[b31] Pedregosa F. *et al.* Scikit-learn: Machine Learning in Python. J. Mach. Learn. Res. 12, 2825–2830 (2011).

[b32] Malley J. D., Kruppa J., Dasgupta A., Malley K. G. & Ziegler A. Probability machines: consistent probability estimation using nonparametric learning machines. Methods Inf Med 51, 74–81 (2012). 21915433 10.3414/ME00-01-0052 PMC3250568

[b33] James G., Witten D. M., Hastie T. & Tibshirani R. in An introduction to statistical learning with applications in R Ch. 9, 337–372 (Springer: New York, , 2013).

[b34] Appavu S., Rajaram R., Nagammai M., Priyanga N. & Priyanka S. in Advances in Computer Science and Information Technology (eds. Meghanathan N., Kaushik B. K. & Nagamalai D.) 501–511 (Springer: Berlin Heidelberg, , 2011).

[b35] Sing T., Sander O., Beerenwinkel N. & Lengauer T. ROCR: visualizing classifier performance in R. Bioinformatics 21, 3940–3941 (2005). 16096348 10.1093/bioinformatics/bti623

[b36] Robin X. *et al.* pROC: an open-source package for R and S + to analyze and compare ROC curves. BMC Bioinformatics 12, 77 (2011). 21414208 10.1186/1471-2105-12-77 PMC3068975

[b37] Hastie T., Tibshirani R. & Friedman J. in The Elements of Statistical Learning: Data Mining, Inference, and Prediction (Springer-Verlag: New York, , 2009).

[b38] Rosenbaum L., Hinselmann G., Jahn A. & Zell A. Interpreting linear support vector machine models with heat map molecule coloring. J. Cheminformatics 3, 11 (2011). 10.1186/1758-2946-3-11 PMC3076244 21439031

[b39] Loken C. *et al.* SciNet: Lessons Learned from Building a Power-efficient Top-20 System and Data Centre. J. Phys. Conf. Ser. 256, 012026 (2010).

