# Supplementary material for: Smoking Gun or Circumstantial Evidence? Comparison of Statistical Learning Methods using Functional Annotations for Prioritizing Risk Variants
Source: Sci Rep. 2015 Aug 24;5:13373. doi: 10.1038/srep13373 (PMC4642511; doi:10.1038/srep13373)
Supplement: Supplementary Information [file srep13373-s1.pdf]

Supplementary information for:

Smoking Gun or Circumstantial Evidence? Comparison of Statistical Learning Methods using Functional Annotations for Prioritizing Risk Variants

Sarah A Gagliano, Reena Ravji, Michael R Barnes, Michael E Weale and Jo Knight

Supplementary Table 1. Summary statistics of the prediction score distributions for the various models based on the GWAS Catalogue classifier. For a visual representation see the violin plots (Fig. 1). [SD=standard deviation]

Functional Annotations		Gagliano et al.		Ritchie et al.		Kircher et al.	
		Hits	Non-hits	Hits	Non-hits	Hits	Non-hits
Elastic Net (not scaled)	Minimum	0.32	0.32	0.36	0.34	0.22	0.14
	Median	0.54	0.44	0.49	0.44	0.54	0.41
	Mean	0.54	0.46	0.52	0.47	0.55	0.43
	Maximum	0.92	0.93	0.89	0.91	0.93	0.93
	SD	0.13	0.12	0.11	0.09	0.15	0.15
Random Forest (altered minimum node size)	Minimum	0.12	0.12	0.23	0.21	0.21	0.16
	Median	0.55	0.44	0.55	0.43	0.53	0.44
	Mean	0.54	0.46	0.53	0.45	0.42	0.43
	Maximum	0.88	0.88	0.75	0.76	0.83	0.84
	SD	0.13	0.12	0.12	0.13	0.12	0.14
Support Vector Machine (with prior feature selection)	Minimum	0.33	0.33	0.43	0.43	0.18	0.09
	Median	0.61	0.49	0.48	0.44	0.52	0.44
	Mean	0.58	0.50	0.55	0.49	0.58	0.50
	Maximum	0.91	0.93	1.00	1.00	0.98	0.99
	SD	0.14	0.14	0.15	0.11	0.18	0.14

Supplementary Table 2. Proportion of GWAS Catalogue hits for the various models. Results are shown for the variants in the test set data that were assigned the highest prediction scores (top quartile) and the lowest scored variants (lower quartile). The difference row shown corresponds to the proportion of GWAS significant variants in the top quartile minus that of the lower quartile, so a positive difference suggests that the quartile of the most highly scored variants (top quartile) contains more GWAS significant variants compared to the lowest scored variants (lower quartile). The number of variants present in each quartile are in parentheses. Note that quartiles can vary in size where prediction scores are identical across many variants, and all those variants with that particular score were included in the quartile.

Annotation set						
	Gagliano et al.		Ritchie et al.		Kircher et al.	
	Elastic Net					
		Chi-sq p-val		Chi-sq p-val		Chi-sq p-val
top quartile	8.8% (7872)		7.4% (7823)		10% (2656)	
lower quartile	2.2% (8261)	< 2.2e-16	2.1% (7837)	< 2.2e-16	1.1% (2655)	< 2.2e-16
Difference	6.6%		5.3%		9.3%	
	Random Forest					
		Chi-sq p-val		Chi-sq p-val		Chi-sq p-val
top quartile	8.8% (7956)		7.8% (7826)		10% (2654)	
lower quartile	2.2% (7889)	< 2.2e-16	1.4% (7825)	< 2.2e-16	1.0% (2654)	< 2.2e-16
Difference	6.6%		6.4%		9.1%	
	Support Vector Machine					
		Chi-sq p-val		Chi-sq p-val		Chi-sq p-val
top quartile	8.1% (7873)		7.3% (8150)		8.1% (2655)	
lower quartile	2.2% (7807)	< 2.2e-16	2.2% (7555)	< 2.2e-16	2.9% (2654)	< 2.2e-16
Difference	5.8%		5.1%		5.2%	

Supplementary Table 3. Pairwise correlation between prediction scores in the test set between models either holding the annotation set or the algorithm constant in the primary analysis. EN= elastic net, RF=random forest, SVM= support vector machine

			Annotation set								
			Gagliano et al.			Ritchie et al.			Kircher et al.		
		Algorithm	EN	RF	SVM	EN	RF	SVM	EN	RF	SVM
Annotation set	Gagliano et al.	EN	--	0.95	0.98	0.41	--	--	0.47	--	--
		RF	0.95	--	0.93	--	0.47	--	--	0.51	--
		SVM	0.98	0.93	--	--	--	0.28	--	--	0.35
	Ritchie et al.	EN	0.41	--	--	--	0.84	0.79	0.71	--	--
		RF	--	0.47	--	0.84	--	0.66	--	0.82	--
		SVM	--	--	0.28	0.79	0.66	--	--	--	0.69
	Kircher et al.	EN	0.47	--	--	0.71	--	--	--	0.84	0.72
		RF	--	0.51	--	--	0.82	--	0.84	--	0.69
		SVM	--	--	0.35	--	--	0.69	0.72	0.69	--

Supplementary Table 4. Proportion of sub-genome-wide-significant variants ($5x10^{-8}<p<1x10^{-6}$) variants from the first round of the schizophrenia GWAS (PGC1) that are GWAS significant (p<5e-8) in the second round (PGC2) for the various models. Results are shown for the variants that were assigned the highest scores (top quartile) and the lowest scored variants (lower quartile). The difference row shown corresponds to the proportion of GWAS significant variants in the top quartile minus that of the lower quartile, so a positive difference suggests that the quartile of the most highly scored PGC1 sub-genome-wide significant variants (top quartile) contains more GWAS significant variants from PGC2 compared to the lowest scored PGC1 sub-genome-wide significant variants (lower quartile). The number of variants present in each quartile are in parentheses. Note that quartiles can vary in size where prediction scores are identical across many variants, and all those variants with that particular score were included in the quartile.

Annotation set						
	Gagliano et al.		Ritchie et al.		Kircher et al.	
	Elastic Net					
		Chi-sq p-val		Chi-sq p-val		Chi-sq p-val
top quartile	83% (60)		77% (56)		54% (34)	
lower quartile	79% (66)	0.52	55% (56)	0.02	43% (37)	7.30E-05
Difference	4%		22%		11%	
	Random Forest					
		Chi-sq p-val		Chi-sq p-val		Chi-sq p-val
top quartile	65% (60)		72% (55)		71% (41)	
lower quartile	90% (59)	1.20E-03	51% (55)	0.02	53% (43)	0.10
Difference	-25%		21%		18%	
	Support Vector Machine					
		Chi-sq p-val		Chi-sq p-val		Chi-sq p-val
top quartile	50% (54)		70% (56)		73% (37)	
lower quartile	79% (68)	6.30E-04	67% (52)	0.79	64% (42)	0.41
Difference	-29%		3%		9%	

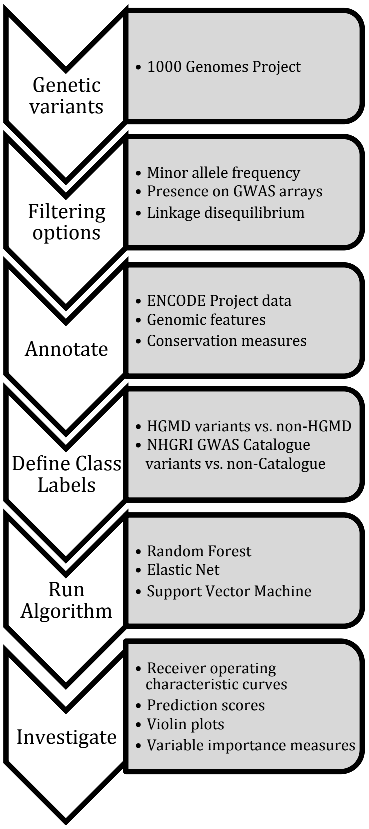

Supplementary Figure 1. Various steps in the statistical learning pipeline for genetic variant prioritization using functional annotations, with examples outlined for each; GWAS=Genome-wide association studies; ENCODE= Encyclopedia of DNA Elements; NHGRI= National Human Genome Research Institute; HGMD= Human Gene Mutation Database

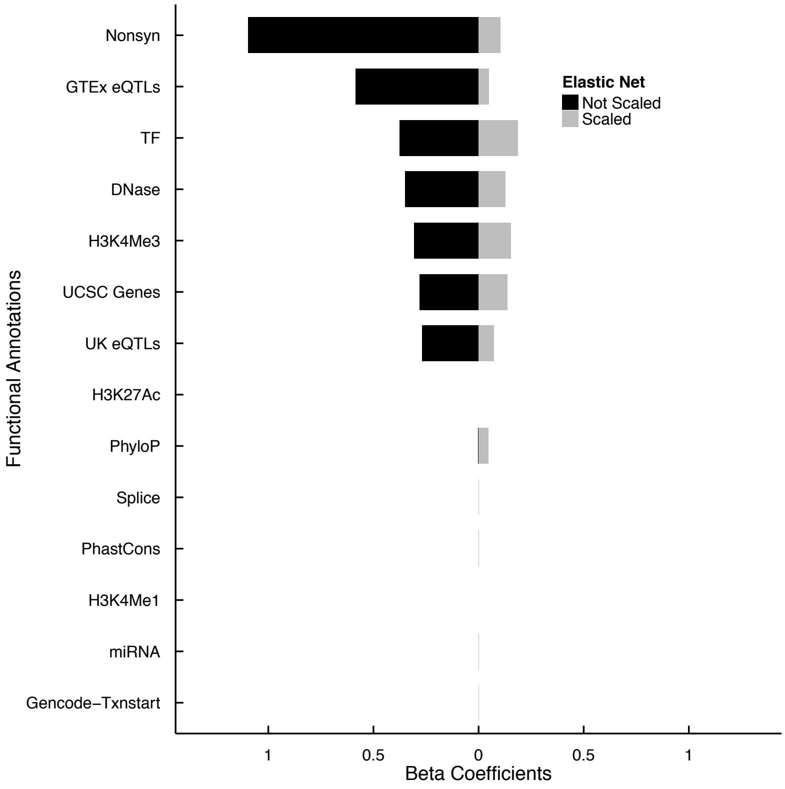

Supplementary Figure 2. Feature importance for models using the Gagliano et al. annotations based on the GWAS Catalogue classifier. The importance of annotations differed when using scaled versus non-scaled annotations in elastic net [splice= splice sites, Nonsyn= nonsynonymous SNPs, DNase= DNase I hypersensitive sites, GTEx eQTLs= cis-eQTL data from the GTEx Consortium, UK eQTLs= cis-eQTL data from the UK Brain Consortium, Phylo= PhyloP conservation, PhastCons= PhastCons conservation, H3K4MeMe1= H3K4Me1 histone modification, H3K4Me3= H3K4Me3 histone modification, H3K27Ac=H3K27Ac histone modification, TF= transcription factor binding sites, miRNA= micro RNA targets, Gencode-Txnstart= transcription start sites from Gencode]

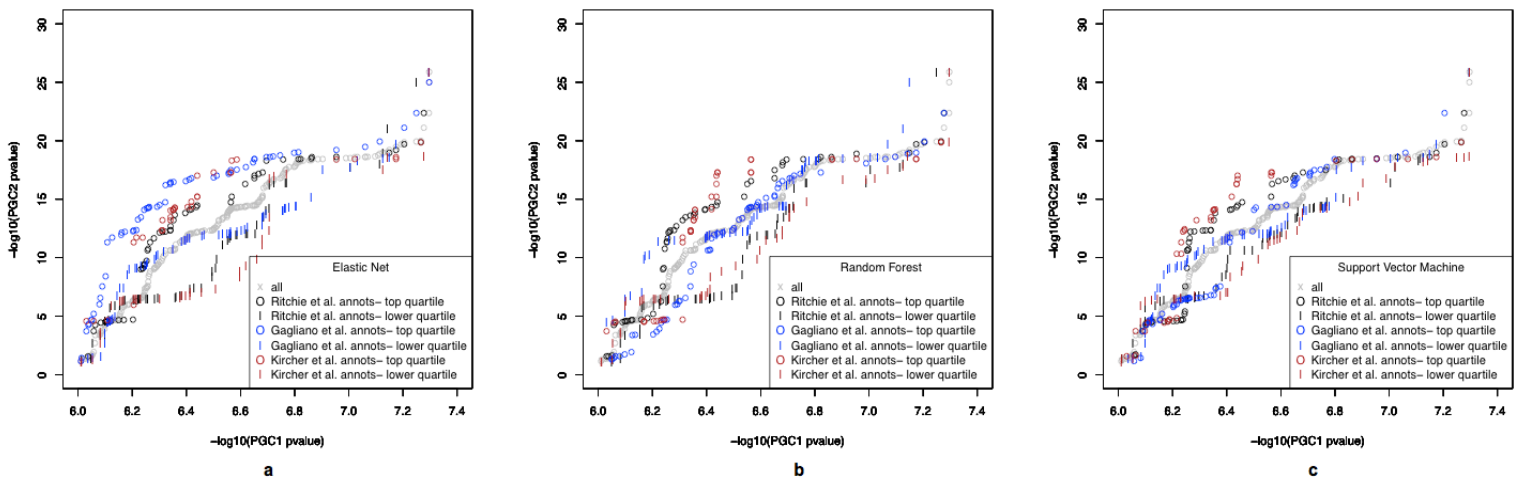

Supplementary Figure 3. Quantile-quantile plots of PGC1 sub-genome-wide-significant variants ($5x10^{-8}<p<1x10^{-6}$) stratified by prediction scores for the various models based on the GWAS Catalogue classifier, and plotted by -log10(PGC1 p-values) versus -log10(PGC2 p-values). Models grouped by algorithm: elastic net (non-scaled annotations) [**a**], random forest (adjusted minimum node size) [**b**], and support vector machine (with prior feature selection) [**c**]. The lower quartile genetic variants are those PGC1 sub-genome-wide significant variants that were assigned the lowest prediction scores (in the first quartile), and the top quartile variants are those with the highest prediction scores (in the fourth quartile).

Supplementary Text

Computational time

All of the models run in this paper took under 130 minutes to complete (**Supplementary Tables 5-7**). Note that for the support vector machine, in addition to the linear kernel, we also tried using the radial basis function kernel (the type of kernel one step more complex than linear). The time limiting step is conducting a “gridsearch” (running through different values of the generalization parameter C until convergence is reached). We could not achieve convergence using the radial basis function kernel within a reasonable amount of time (ie. still no convergence after running 48 hours on a high performance computing cluster).

Supplementary Table 5. Time it takes for the GWAS Catalogue comparisons, holding data and classifier constant, while varying algorithm and annotations.

**Annotations →**	**Gagliano et al.**	**Ritchie et al.**	**Kircher et al.**
Elastic Net	6m20s	47m10s	105m0s
Random Forest (altered minimum node size)	0m6s	0m39s	0m68s
Support Vector Machine (with prior feature selection)	0m17s	0m8s	0m4s

Supplementary Table 6. Time it takes for the HGMD comparisons, holding data and classifier constant, while varying algorithm and annotations.

**Annotations →**	**Gagliano et al.**	**Ritchie et al.**	**Kircher et al.**
Elastic Net	2m35s	18m24s	129m2s
Random Forest (altered minimum node size)	0m3s	0m14s	0m82s
Support Vector Machine (with prior feature selection)	0m8s	0m7s	0m11s

Supplementary Table 7. Time it takes for the non-exonic HGMD comparisons, holding data and classifier constant, while varying algorithm and annotations.

**Annotations →**	**Gagliano et al.**	**Ritchie et al.**	**Kircher et al.**
Elastic Net	1m30s	25m29s	49m20s
Random Forest (altered minimum node size)	0m2s	0m9s	0m42s
Support Vector Machine (with prior feature selection)	0m2s	0m3s	0m2s

Comparison of scores from the three papers: Application to Schizophrenia GWAS

Methods

In the effort for a more general comparison of the published methods as is, rather than looking specifically at the algorithm and annotations, we additionally conducted the schizophrenia GWAS application using scores for the variants obtained directly from the published papers. Gagliano et al. makes available prediction scores from the non-phenotype specific analysis (which defined risk variants as variants present in the NHGRI GWAS Catalogue[13] downloaded on August 6, 2013 with a p-value of less than or equal to $5x10^{-8}$, and controls as variants on common GWAS platforms that are not in linkage disequilibrium (r^2>=0.8) with the GWAS Catalogue variants). Ritchie et al. makes available prediction scores from three models. We used the most stringent, the scores from the "region" model (which defined risk variants as "regulatory mutations" in the Human Gene Mutation Database (HGMD)[15] public database, and the control variants as all those variants in the 1000 Genomes Project within a kilobase distance from each HGMD variant. Regulatory mutations are those variants that fall into regions that do not encode for a protein. For both Gagliano et al. and Ritchie et al. the prediction scores range from 0 to 1, where a value closer to one assigned to a variant suggests that that variant is more likely to be a risk variant as defined in the models. Kircher et al. defined phred-like scores (scaled C scores) in addition to raw scores. We plotted based on the raw scores.

Results

When using the actual prediction scores made available in the three papers, the quantile-quantile plot suggested that the Gagliano et al. scores best identified the novel hits from the second round of the schizophrenia GWAS that were not significant in the first round (**Supplementary Fig. 4**). The proportion of hits in the top versus the bottom quartiles of prediction scores are significantly different for the Gagliano et al. method (p<0.03, chi-square test), whereas the difference between the quartiles for the Ritchie et al. and Kircher et al. methods were not significant (p~0.4 for both methods) (**Supplementary Table 8**).

Of the variants in the top quartile for the Gagliano et al. scores, most (80%) were GWAS significant variants ($p<5x10^{-8}$) from the second round of the GWAS. Of the variants in the top quartile for the Ritchie et al. scores and the Kircher et al. scores there were less significant variants: 67% and 74% respectively. Only a small percentage of variants in the top quartiles were nonsynonymous SNPs (ie. missense, nonsense, frameshift, inframe indel, or stop-lost mutations): 9%, 2% and 4% for the Gagliano et al. scores, Ritchie et al. scores and Kircher et al. scores, respectively. Of the sub-genome-wide significant PGC1 SNPs, only 5% are nonsynonymous, and of those, most (83%) become PGC2 hits.

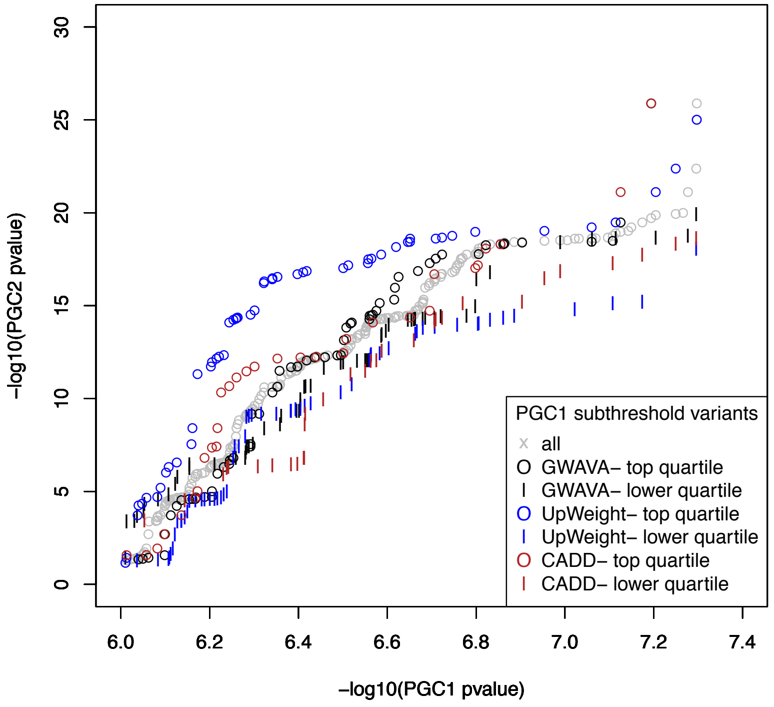

Supplementary Figure 4. Quantile-quantile plots of PGC1 sub-genome-wide-significant variants ($5x10^{-8}<p<1x10^{-6}$) stratified by prediction scores obtained from the three papers, and plotted by -log10(PGC1 p-values) versus -log10(PGC2 p-values). "GWAVA" corresponds to the scores obtained from the method published in Ritchie et al. 2014, "UpWeight" corresponds to the method in Gagliano et al. 2014 and "CADD" corresponds to the method in Kircher et al. 2014. The lower quartile genetic variants are those with a prediction score in the first quartile, and the top quartile variants are those with prediction values in the fourth quartile.

Supplementary Table 8. Using the scores from the actual published models, the proportion of sub-genome-wide-significant variants ($5x10^{-8}<p<1x10^{-6}$) variants from the first round of the schizophrenia GWAS (PGC1) that are GWAS significant (p<5e-8) in the second round (PGC2) for the various models. Results are shown for the variants that were assigned the highest scores (top quartile) and the lowest scored variants (lower quartile). The difference row shown corresponds to the proportion of GWAS significant variants in the top quartile minus that of the lower quartile, so a positive difference suggests that the quartile of the most highly scored PGC1 sub-genome-wide significant variants (top quartile) contains more GWAS significant variants from PGC2 compared to the lowest scored PGC1 sub-genome-wide significant variants (lower quartile). The number of variants present in each quartile are in parentheses. Note that quartiles can vary in size where prediction scores are identical across many variants, and all those variants with that particular score were included in the quartile. “UpWeight” corresponds to the method in Gagliano et al. 2014, “GWAVA” corresponds to the scores obtained from the method published in Ritchie et al. 2014, and “CADD” corresponds to the method in Kircher et al. 2014.

	Method					
	UpWeight		GWAVA		CADD	
		Chi-sq p-val		Chi-sq p-val		Chi-sq p-val
top quartile	80% (55)	0.03	67% (60)	0.48	74% (31)	0.41
lower quartile	61% (59)		73% (62)		65% (31)	
Difference	19%		-6%		9%	

Supplementary Tables 9-11 and Supplementary Figures 5-7:

These models are based on the following classifier: variants in the GWAS Catalogue with a p-value < 5E-8 and a random subset of control variants from common genotyping arrays. The annotations from Gagliano et al. were used.

Supplementary Table 9. Importance of annotations by Elastic Net

Nonsynonymous	1.09457232
GTEx_eQTLs	0.58352715
TFBS	0.37469007
DNase_I	0.34800772
H3K4Me3	0.30673014
UCSC_Genes	0.2806802
UK_Brain_eQTLs	0.26813544
H3K27Ac	0.11850658
PhyloP	0.00121327
Splice	0
PhastCons	0
H3K4Me1	0
miRNA	0
Gencode_Txnstart	0

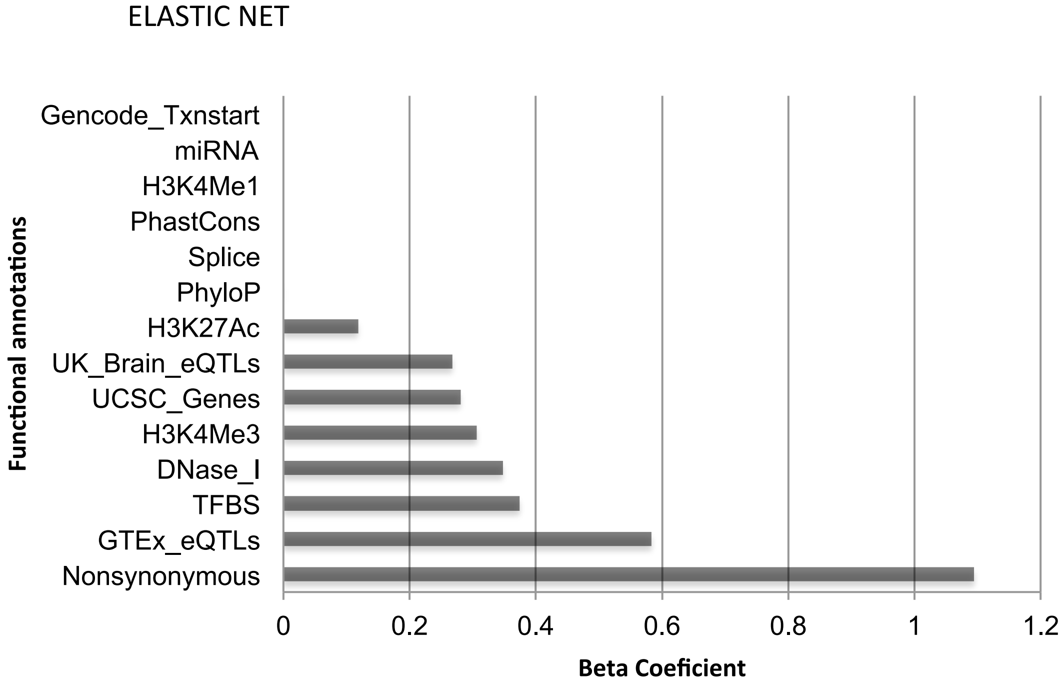

Supplementary Figure 5. Importance of annotations by Elastic Net

Supplementary Table 10. Importance of annotations by Random Forest

H3K4Me3	0.16194218
TFBS	0.16076837
H3K27ac	0.11930863
UCSC	0.11125026
DNase_I	0.09617033
PhyloP	0.08126218
Nonsynonymous	0.07904821
PhastCons	0.06953112
H3K4Me1	0.04671044
UK_Brain_eQTLs	0.04111495
GTEx_eWTLs	0.0287619
Splice	0.0027309
Gencode_Txnstart	0.00094908
miRNA	0.00045145

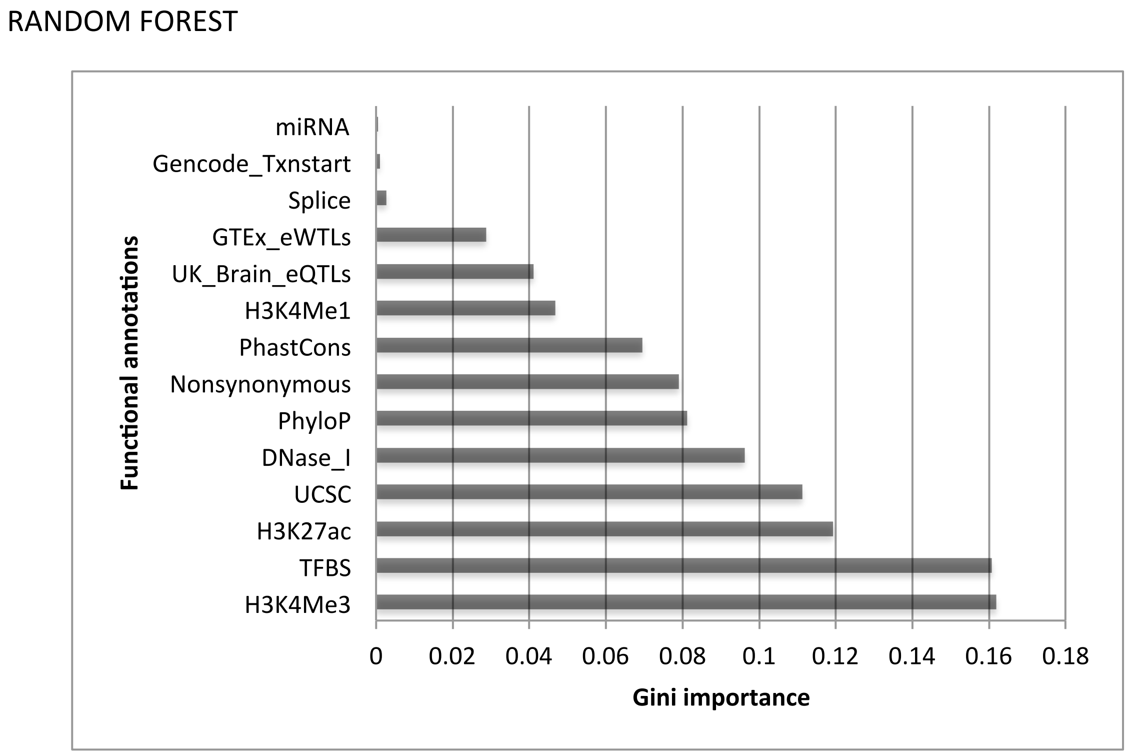

Supplementary Figure 6. Importance of annotations by Random Forest

Supplementary Table 11. Importance of annotations by Support Vector Machine

TFBS	0.30471134
H3K4Me3	0.27845552
H3K27ac	0.1374529
DNase_I	0.13058098
Nonsynonymous	0.12064785
UCSC_Genes	0.11260425
UK_Brain_eQTLs	0.07259671
GTEx_eQTLs	0.0394731
PhyloP	0.03845896

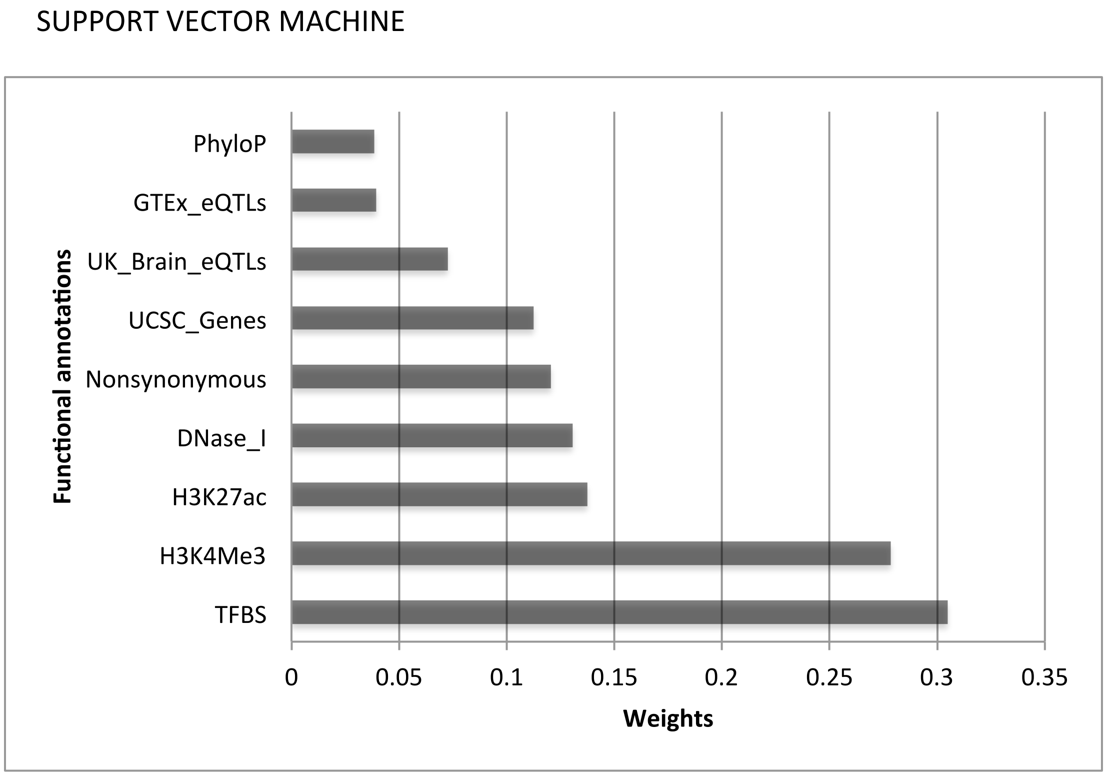

Supplementary Figure 7. Importance of annotations by Support Vector Machine

For Supplementary Tables 9-11 and Supplementary Figures 5-7:

Annotation Legend: See Gagliano et al. for further details.

Nonsynonymous= Nonsynonymous SNP
GTEx_eQTLs= cis eQTL from the GTEx Project
TFBS= Transcription factor binding site
DNase_I= DNaseI hypersensitive site
H3K4Me3= H4K4Me3 histone modification
UCSC_Genes= UCSC Gene
UK_Brain_eQTLs= cis eQTL from the UK Brain Consortium
H3K27Ac= H3K27Ac histone modification
PhyloP= PhyloP conservation score
Splice= +/-5 base pairs from a splice site
PhastCons= PhastCons conservation score
H3K4Me1= H3K4Me1 histone modification
miRNA= microRNA target as defined by TargetScan
Gencode_Txnstart= Transcription start site as defined by Gencode

Supplementary Tables 12-14 and Supplementary Figures 8-10:

These models are based on the following classifier: variants in the GWAS Catalogue with a p-value < 5E-8 and a random subset of control variants from common genotyping arrays. The annotations from Ritchie et al. were used.

Supplementary Table 12. Importance of annotations by Elastic Net

CDS	0.73835991
%GC	0.40444405
MEF2A	0.28525379
INTRON	0.12824043
H3K4me1	0.0787302
FOXA2	0.07059569
FOXA1	0.0605902
Average_GERP	0.02813259
H3K27me3	0.02267571
WEAK_ENH	0.02244694
seq_A	0.02225467
bound_motifs	0.01886099

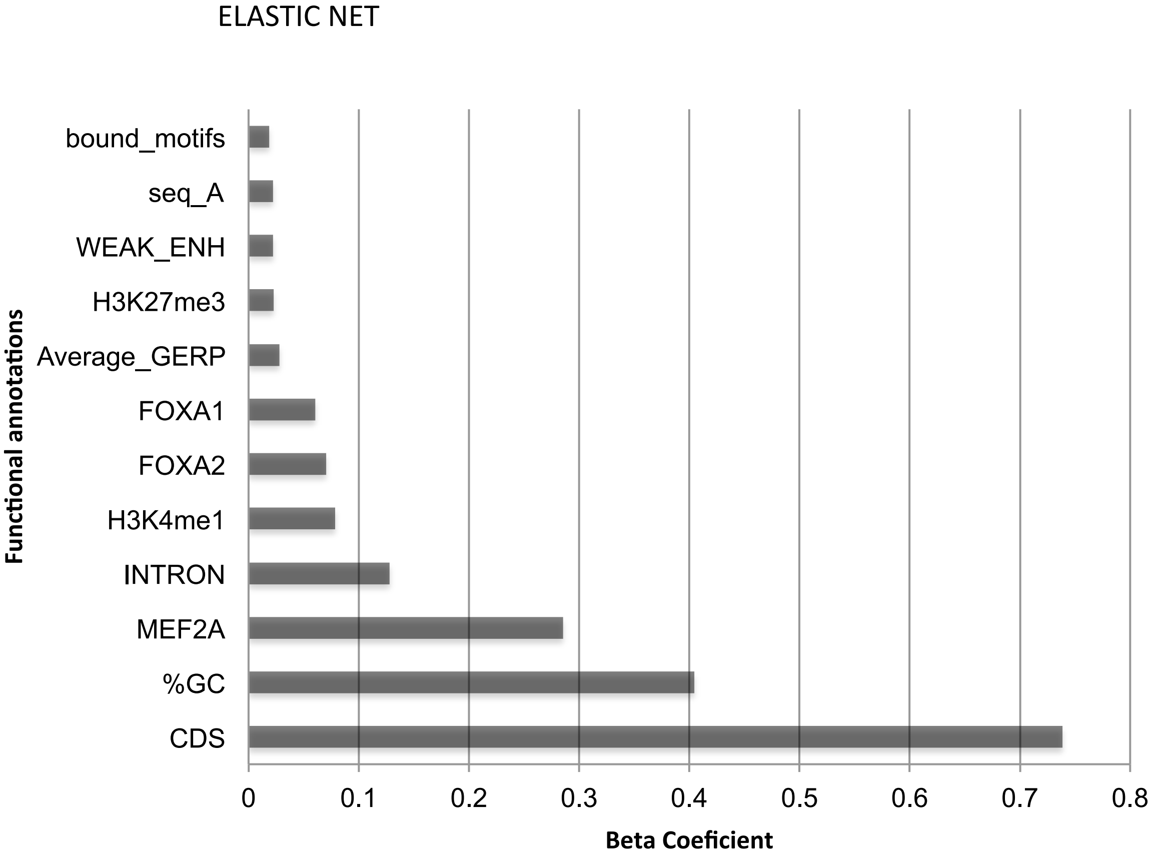

Supplementary Figure 8. Importance of annotations by Elastic Net

Supplementary Table 13. Importance of annotations by Random Forest

SS_distance	0.10484422
H3K4me1	0.10242725
REP	0.09179542
TSS_distance	0.0758117
H3K36me3	0.04611345
H3K9ac	0.04144153
H3K27ac	0.03827775
Average_GERP	0.03809685
%GC	0.03564533
Average_het	0.0350499
CDS	0.03418113
TRAN	0.0332855
Average_DAF	0.02864317
GERP	0.02700529
DNase	0.02631869
H3K79me2	0.02315817
H3K4me3	0.02233333
EXON	0.02109917
H3K4me2	0.0166956
H2AFZ	0.01635564
FAIRE	0.0141235
H3K27me3	0.01177772
dnase_fps	0.00912907
ENH	0.00799977
repeat.	0.00797199
POLR2A	0.00793303
TSS	0.00564932
INTRON	0.00556233
bound_motifs	0.00531464
UTR3	0.00446204

RANDOM FOREST --> too many non-zero annots for a graph (131). The top 30 are displayed here

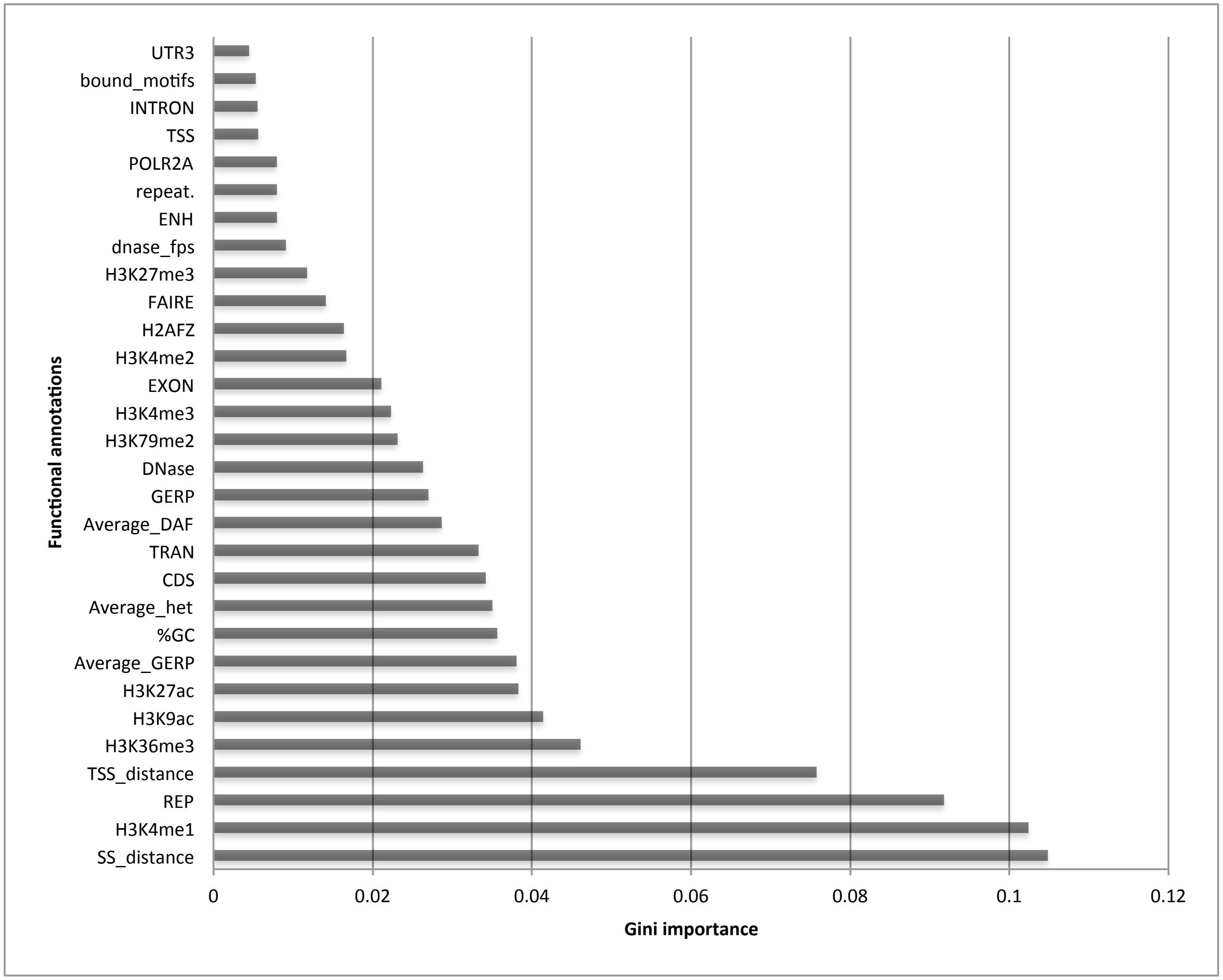

Supplementary Figure 9. Importance of annotations by Random Forest

Supplementary Table 14. Importance of annotations by Support Vector Machine

H3K4me1	0.61016666
CDS	0.42464906
bound_motifs	0.22274777
H3K27me3	0.16459611
WEAK_ENH	0.15813353
MEF2A	0.11712527
FOXA1	0.09599154
FOXA2	0.00525437
%GC	0.00010008
Average_GERP	6.13E-05
INTRON	5.17E-05
seq_A	4.37E-05

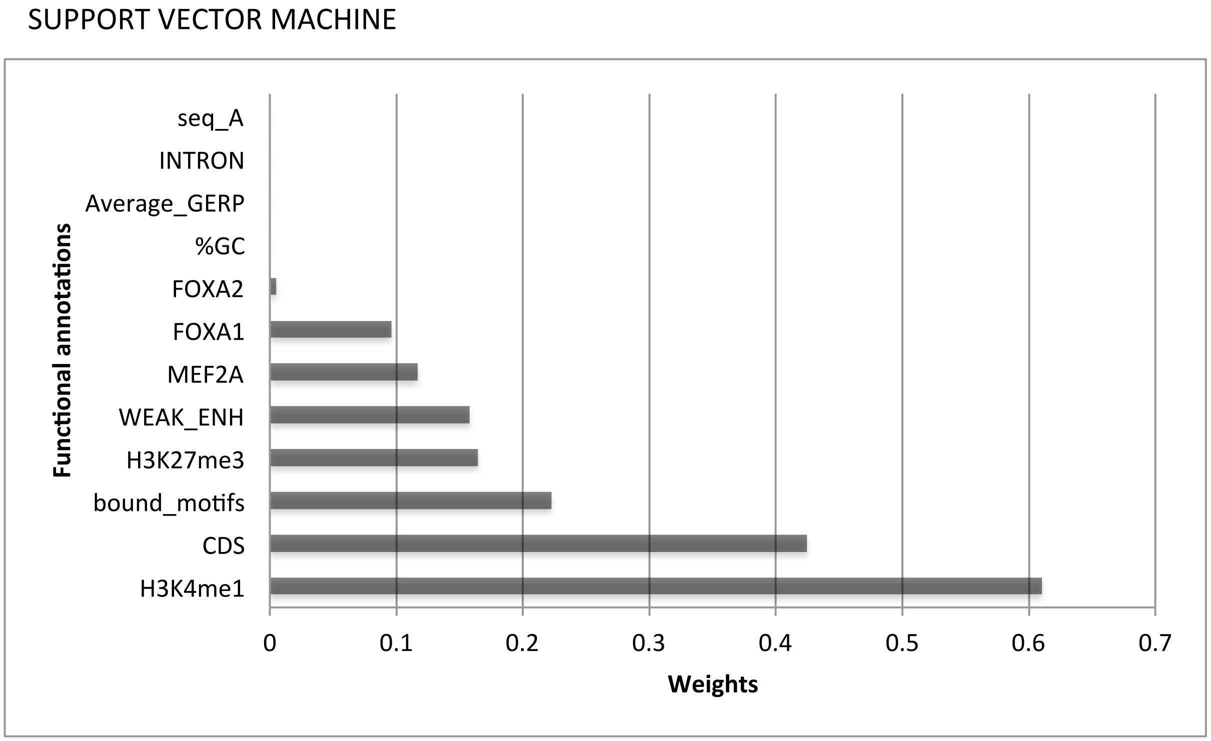

Supplementary Figure 10. Importance of annotations by Support Vector Machine

For Supplementary Tables 12-14 and Supplementary Figures 8-10:

Annotation Legend: See Ritchie et al. for further details.

%GC= GC content of 100bp flanking region

Average_DAF= mean derived allele frequency of variants in 1kb flanking region

Average_GERP= mean GERP score of 100bp flanking region

Average_het= mean heterozygosity of 1kb flanking region

CDS= coding sequence

DNase= DNase1-seq peak

ENH= predicted enhancer segment
EXON= exonic region
FAIRE= FAIRE-seq peak
FOXA1= FOXA1 Transcription Factor ChIP-seq peaks
FOXA2= FOXA2 Transcription Factor ChIP-seq peaks
GC= GC content of 100bp flanking region
GERP= GERP score at the variant locus
H2AFZ= H2AFZ Histone modification ChIP-seq peaks
H3K27ac= H3K27ac Histone modification ChIP-seq peaks
H3K27me3= H3K27me3 Histone modification ChIP-seq peaks
H3K36me3= H3K36me3 Histone modification ChIP-seq peaks
H3K4me1= H3K4me1 Histone modification ChIP-seq peaks
H3K4me2= H3K4me2 Histone modification ChIP-seq peaks
H3K4me3= H3K4me3 Histone modification ChIP-seq peaks
H3K79me2= H3K79me2 Histone modification ChIP-seq peaks
H3K9ac= H3K9ac Histone modification ChIP-seq peaks
INTRON= intronic region
MEF2A= MEF2A Transcription Factor ChIP-seq peaks
POLR2A= POLR2A Transcription Factor ChIP-seq peaks
REP= predicted repressed sequence
SS_distance= distance to the nearest splice site
TRAN= predicted transcribed segment
TSS= predicted promoter segment
TSS_distance= distance to the nearest TSS
UTR3= 3 prime untranslated region
WEAK_ENH= predicted weak enhancer segment
bound_motifs= bound transcription factor motifs
dnase_fps= DNase1-seq footprint
repeat.= annotated repeat element
seq_A= reference base at variant locus is A

Supplementary Tables 15-17 and Supplementary Figures 11-13:

These models are based on the following classifier: variants in the GWAS Catalogue with a p-value < 5E-8 and a random subset of control variants from common genotyping arrays. The annotations from Kircher et al. were used.

Supplementary Table 15. Importance of annotations by Elastic Net

SegwayxR4	0.23214744
NxS	0.18512232
priPhCons	0.1068047
GerpN	0.09977634
RxpriPhCons	0.0898631
SegwayxR3	0.07726528
NSxminDistTSS	0.06787831
RxpriPhyloP	0.05626706
AltxC	0.05350694
NCxGerpS	0.04830807
RxGerpN	0.03203313
RefxA	0.03125206
EncH3K4Me1	0.02614708
CxA	0.00567947
NSxbStatistic	0.00030707
EncH3K4Me3	0.00015327

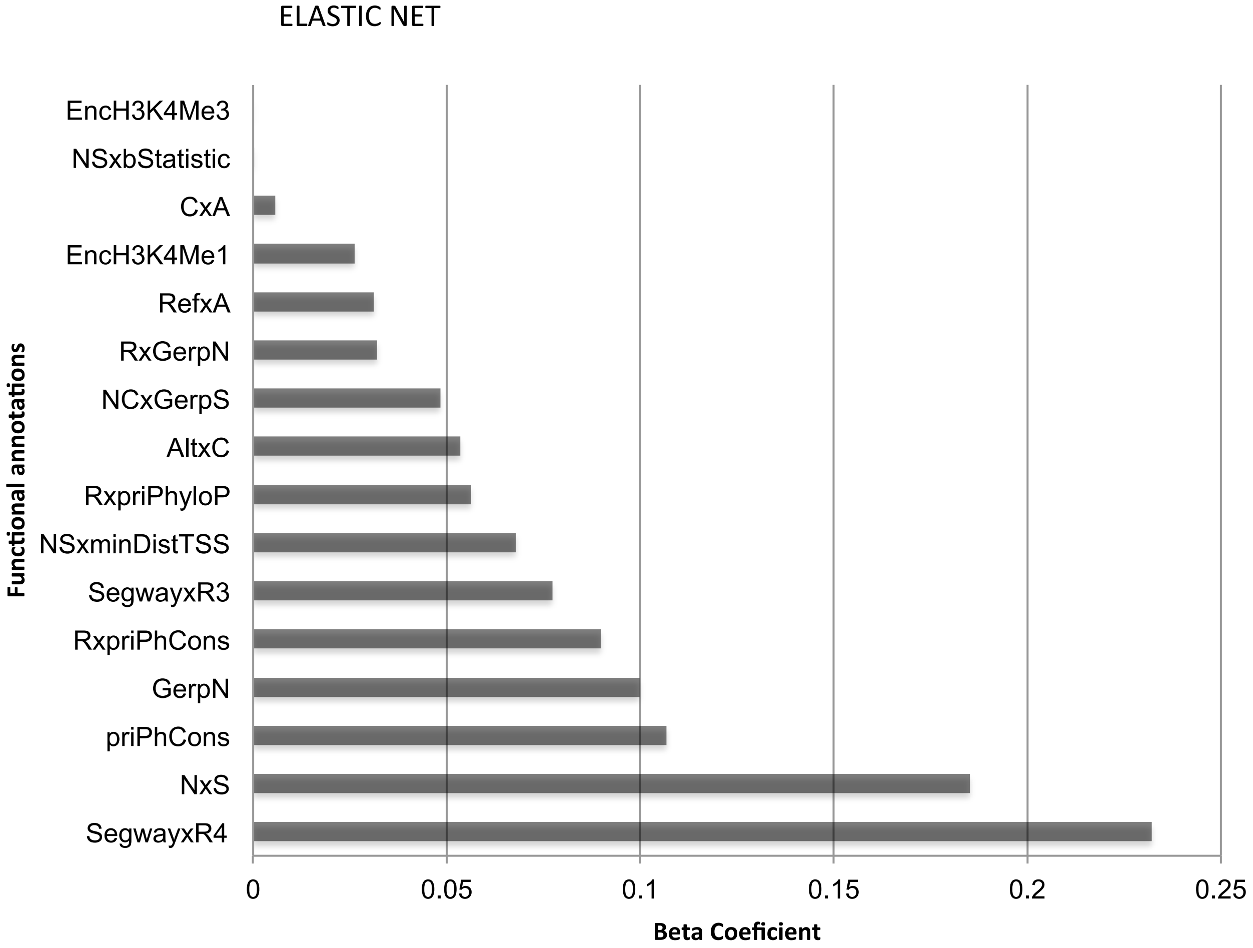

Supplementary Figure 11. Importance of annotations by Elastic Net

Supplementary Table 16. Importance of annotations by Random Forest

bStatistic	0.07612596
EncH3K4Me1	0.041142
minDistTSS	0.03549463
minDistTSE	0.0324394
EncH3K4Me3	0.03210379
IxbStatistic	0.03040545
EncH3K27Ac	0.02770442
GerpN	0.02402458
IGxminDistTSE	0.02248406
EncExp	0.0207314
IGxbStatistic	0.01747853
GerpS	0.01685097
TFBSPeaks	0.01645934
EncOCFaireSig	0.01505465
priPhyloP	0.01321063
IxminDistTSE	0.01247181
TFBSPeaksMax	0.0123973
priPhCons	0.01186039
IxminDistTSS	0.01176682
EncOCmycSig	0.01164866
IGxminDistTSS	0.01163399
EncOCCombPVal	0.01150561
GC	0.01120606
EncOCpolIISig	0.01082852
verPhyloP	0.01070643
TFBS	0.01065238
IGxpriPhCons	0.01062909
EncOCctcfSig	0.01006817
EncOCDNasePVal	0.00883529
EncOCDNaseSig	0.00868473

RANDOM FOREST --> too many non-zero annots for a graph (239). The top 30 are displayed here.

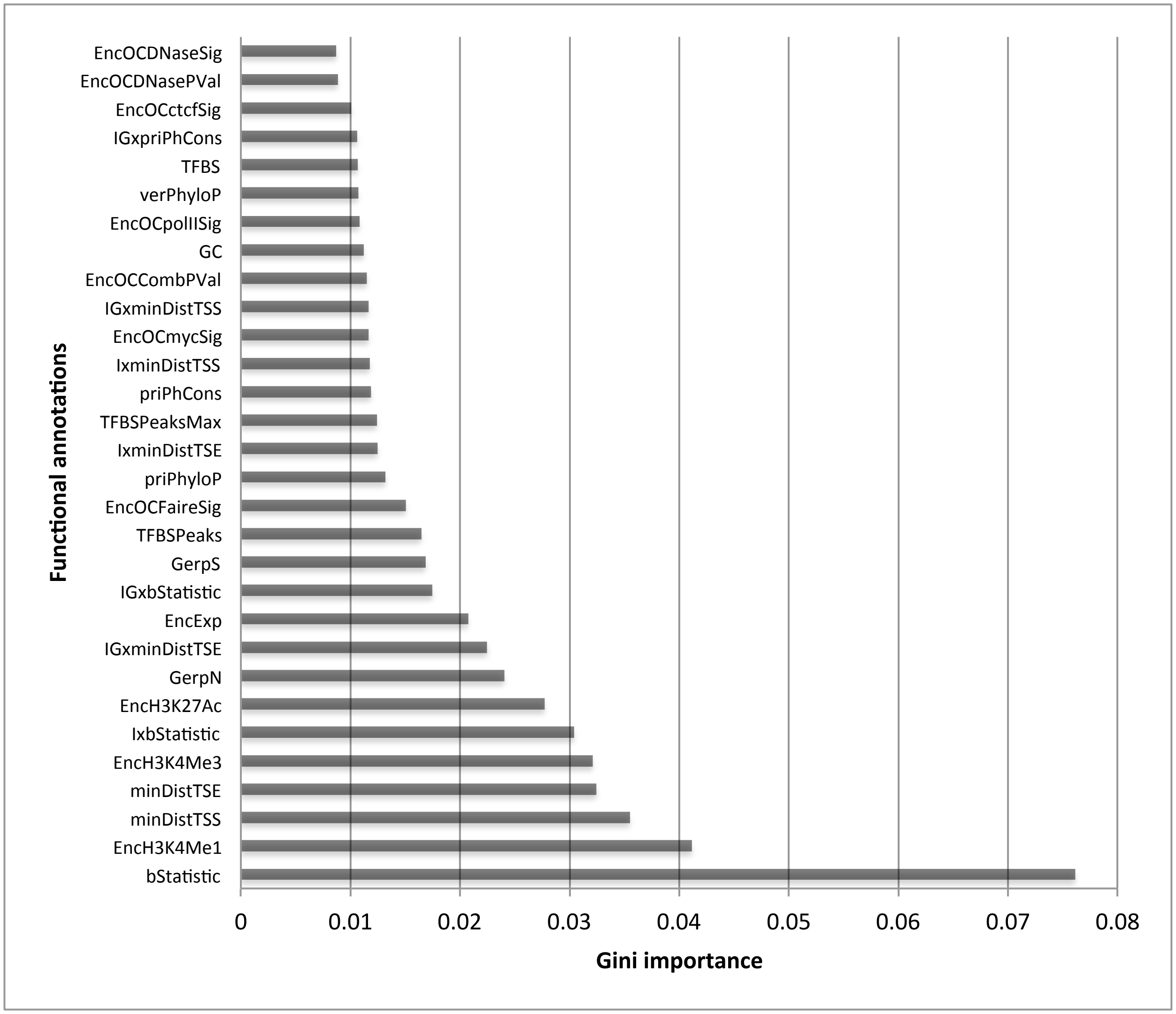

Supplementary Figure 12. Importance of annotations by Random Forest

Supplementary Table 17. Importance of annotations by Support Vector Machine

EncH3K4Me1	0.39204676
EncH3K4Me3	0.19670888
RxGerpN	0.16826805
SegwayxR4	0.15207001
GerpN	0.12315151
NSxminDistTSS	0.11758948
priPhCons	0.10252227
SegwayxR3	0.08543625
NSxbStatistic	0.08159819
AltxC	0.06789917
NCxGerpS	0.06358947
RefxA	0.04072076
RxpriPhCons	0.03338482
RxpriPhyloP	0.03325345
CxA	0.02117921
NxS	0.01230448

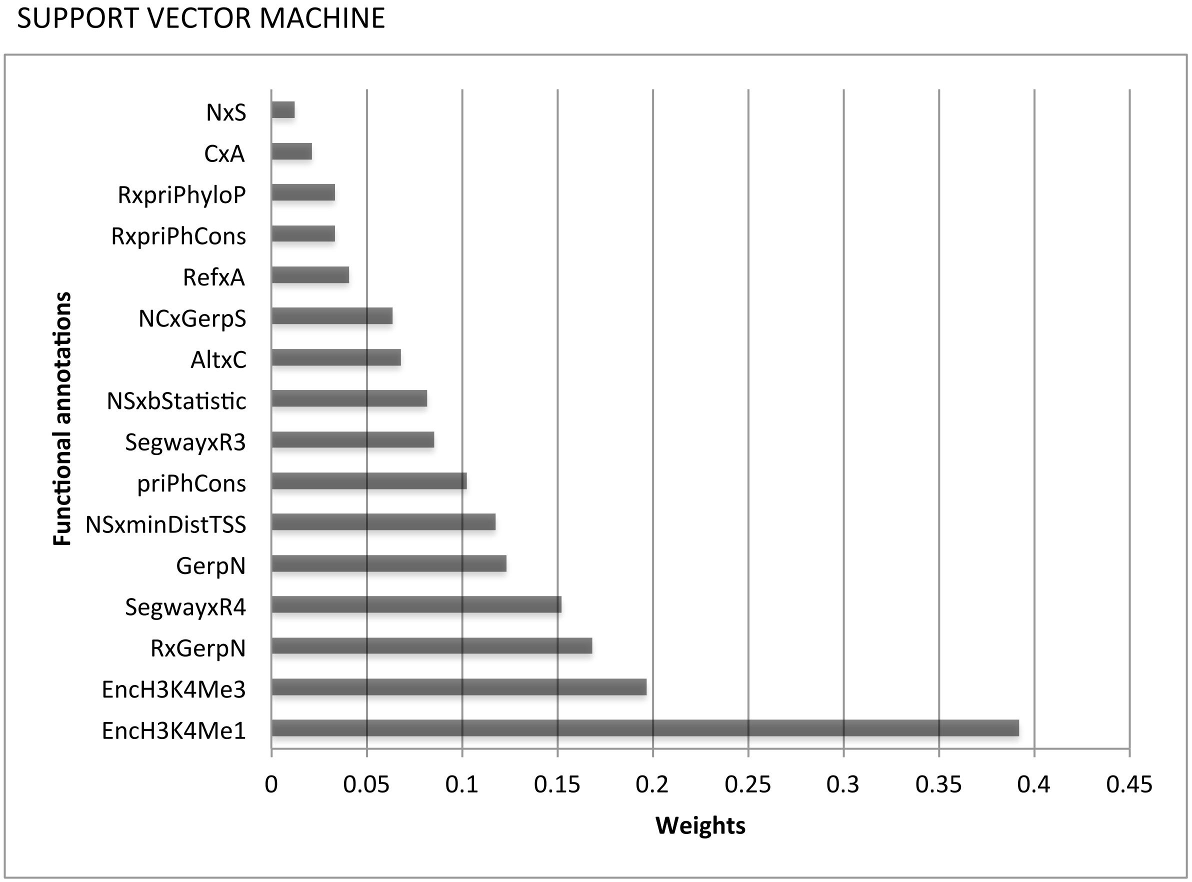

Supplementary Figure 13. Importance of annotations by Support Vector Machine

For Supplementary Tables 15-17 and Supplementary Figures 11-13:

Annotation Legend: See Kircher et al. for further details.

AltxC= Interaction between observed allele and the new amino acid cysteine
CxA= Interaction between the previous amino acid cysteine and the new amino acid alanine
EncExp= Maximum ENCODE expression value
EncH3K27Ac= Maximum ENCODE H3K27 acetylation level
EncH3K4Me1= Maximum ENCODE H3K4 methylation level
EncH3K4Me3= Maximum ENCODE H3K4 trimethylation level
EncOCCombPVal= ENCODE combined p-Value (PHRED-scale) of Faire, Dnase, polII, CTCF, Myc evidence for open chromatin
EncOCDNasePVal= p-Value (PHRED-scale) of Dnase evidence for open chromatin
EncOCDNaseSig= Peak signal for Dnase evidence of open chromatin
EncOCFaireSig= Peak signal for Faire evidence of open chromatin
EncOCctcfSig= Peak signal for CTCF evidence of open chromatin
EncOCmycSig= Peak signal for Myc evidence of open chromatin
EncOCpolIISig= Peak signal for polII evidence of open chromatin
GC= Percent GC in a window of +/- 75bp
GerpN= Neutral evolution score defined by GERP++
GerpS= Rejected Substitution' score defined by GERP++
IGxbStatistic= interaction between intergenic and Background selection score
IGxminDistTSE= interaction between intergenic and Distance to closest Transcribed Sequence End (TSE)
IGxminDistTSS= interaction between intergenic and Distance to closest Transcribed Sequence Start (TSS)
IGxpriPhCons= interaction between intergenic and Primate PhastCons conservation score (excl. human)
IxbStatistic= interaction between intronic and Background selection score
IxminDistTSE= interaction between intronic and Distance to closest Transcribed Sequence End (TSE)
IxminDistTSS= interaction between intronic and Distance to closest Transcribed Sequence End (TSE)
NCxGerpS= interaction between noncoding and Rejected Substitution' score defined by GERP++
NSxbStatistic= interaction between nonsynonymous and Background selection score
NSxminDistTSS= interaction between nonsynonymous and Distance to closest Transcribed Sequence End (TSE)
NxS= interaction between previous amino acid aspargine and synonymous
RefxA= interaction between reference allele and the new amino acid alanine
RxGerpN= interaction between previous amino acid arginine and Neutral evolution score defined by GERP++

RxpriPhCons= interaction between previous amino acid arginine and Primate PhastCons conservation score (excl. human)
RxpriPhyloP= interaction between previous amino acid arginine and Primate PhyloP score (excl. human)
SegwayxR3= Result of genomic segmentation algorithm, R3 category
SegwayxR4= Result of genomic segmentation algorithm, R4 category
TFBS= Number of different overlapping ChIP transcription factor binding sites
TFBSpeaks= Number of overlapping ChIP transcription factor binding site peaks summed over different cell types/tissue
TFBSpeaksMax= Maximum value of overlapping ChIP transcription factor binding site peaks across cell types/tissue
bstatistic= Background selection score
minDistTSE= Distance to closest Transcribed Sequence End (TSE)
minDistTSS= Distance to closest Transcribed Sequence Start (TSS)
priPhCons= Primate PhastCons conservation score (excl. human)
priPhyloP= Primate PhyloP score (excl. human)
verPhyloP= Vertebrate PhyloP score (excl. human)

Supplementary Tables 18-20 and Supplementary Figures 14-16:

These models are based on the following classifier: HGMD and control variants within 1KB of the HGMD variant. The annotations from Gagliano et al. were used.

Supplementary Table 18. Importance of annotations by Elastic Net

miRNA	-2.0913612
UCSC_Genes	-0.4987777
Gencode_Txnstart	-0.1735627
H3K27Ac	-0.1485844
H3K4Me3	-0.0402223
splice	0
PhastCons	0.0020501
PhyloP	0.00248018
H3K4Me1	0.03728084
TFBS	0.05476116
UK_Brain_eQTLs	0.13337447
DNase_I	0.33042212
GTEx_eQTLs	0.73931183
nonsynonymous	1.97196766

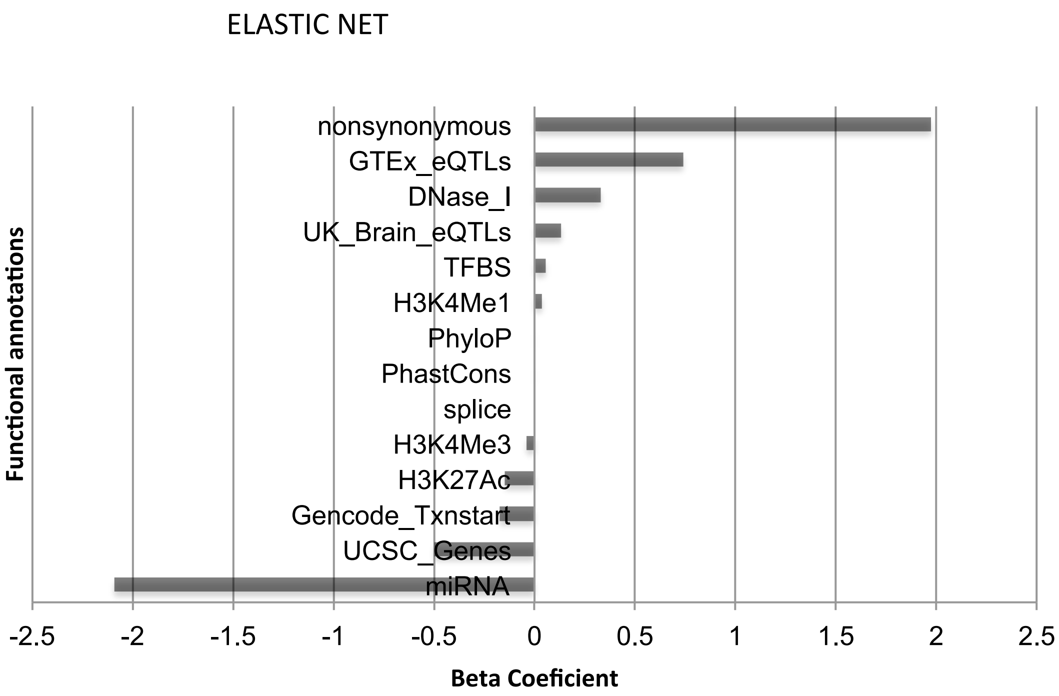

Supplementary Figure 14. Importance of annotations by Elastic Net

Supplementary Table 19. Importance of annotations by Random Forest

nonsynonymous	0.56512023
PhastCons	0.1424801
PhyloP	0.10031994
UCSC_Genes	0.09342726
DNase_I	0.0485705
UK_Brain_eQTLs	0.01121601
H3K4Me1	0.01100313
TFBS	0.00949715
GTEx_eQTLs	0.00874107
H3K27Ac	0.00536329
H3K4Me3	0.0036272
Gencode_Txnstart	0.00052891
miRNA	0.00010522
splice	0

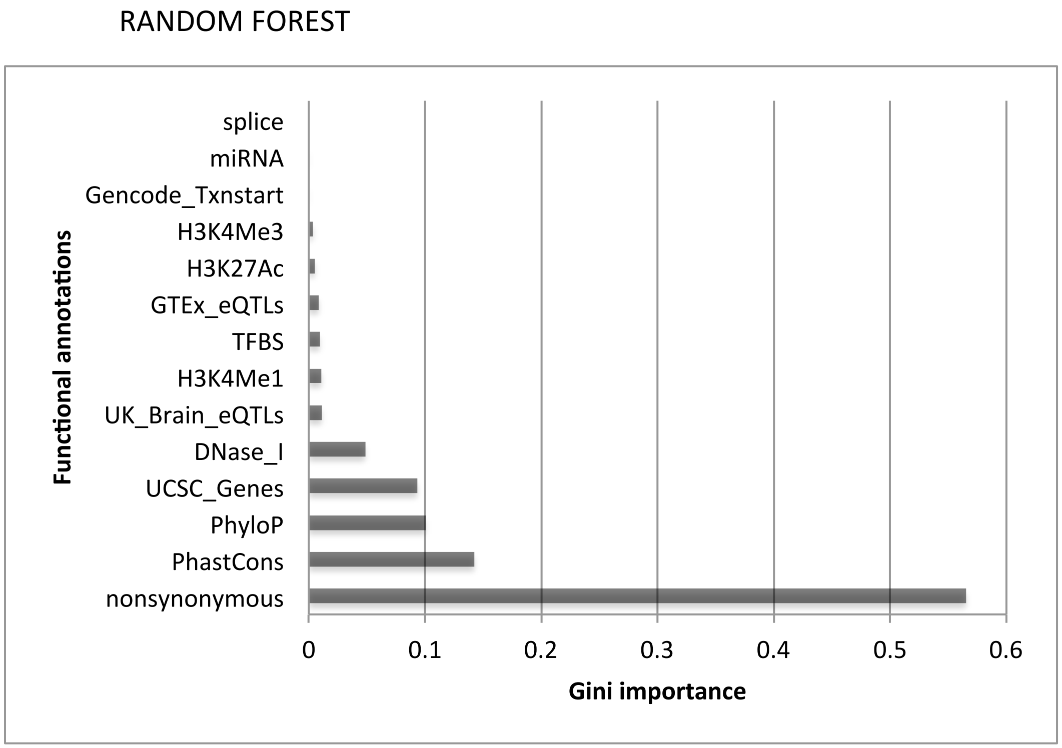

Supplementary Figure 15. Importance of annotations by Random Forest

Supplementary Table 20. Importance of annotations by Support Vector Machine

DNase_I	0.66280725
nonsynonymous	0.60714531
H3K27Ac	0.37433641
GTEx_eQTLs	0.13604832
UK_Brain_eQTLs	0.0001785
PhastCons	3.09E-05
PhyloP	-2.83E-05
UCSC_Genes	-0.0001683

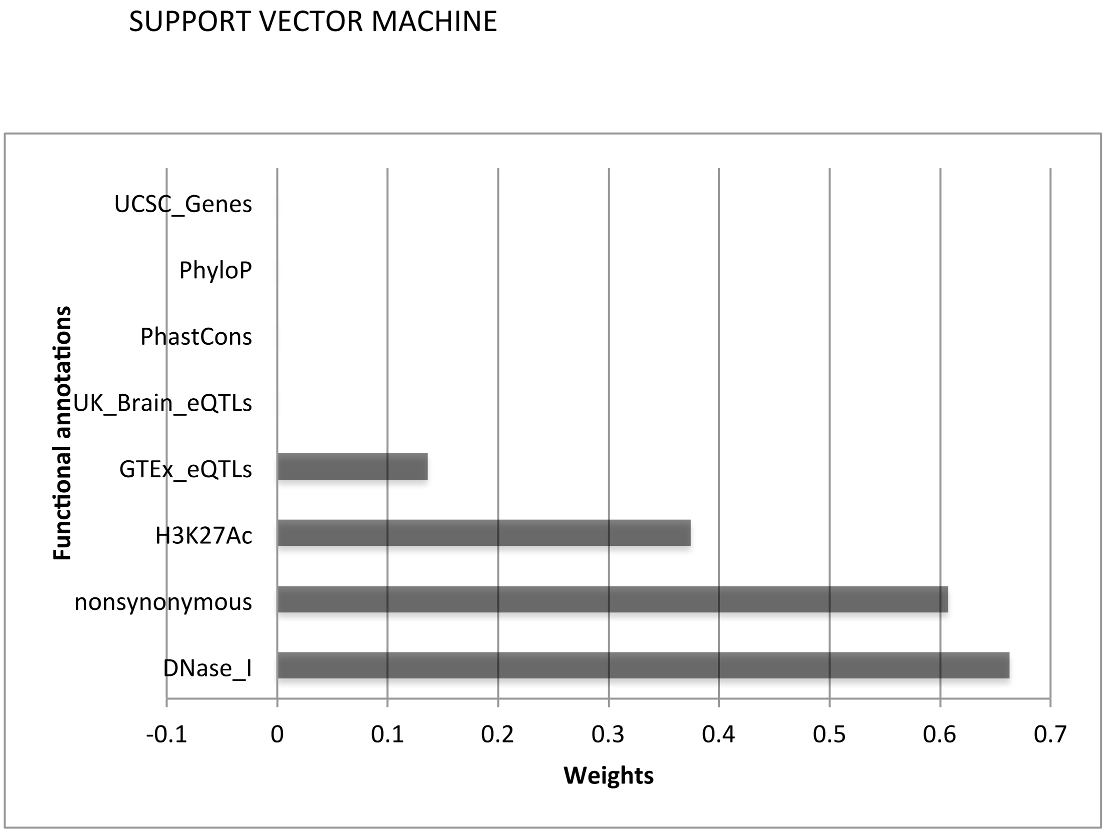

Supplementary Figure 16. Importance of annotations by Support Vector Machine

For Supplementary Tables 18-20 and Supplementary Figures 14-16:

Annotation Legend: See Gagliano et al. for further details.

Nonsynonymous= Nonsynonymous SNP
GTEx_eQTLs= cis eQTL from the GTEx Project
TFBS= Transcription factor binding site
DNase_I= DNaseI hypersensitive site
H3K4Me3= H4K4Me3 histone modification
UCSC_Genes= UCSC Gene
UK_Brain_eQTLs= cis eQTL from the UK Brain Consortium
H3K27Ac= H3K27Ac histone modification
PhyloP= PhyloP conservation score
Splice= +/-5 base pairs from a splice site
PhastCons= PhastCons conservation score
H3K4Me1= H3K4Me1 histone modification
miRNA= microRNA target as defined by TargetScan
Gencode_Txnstart= Transcription start site as defined by Gencode

Supplementary Tables 21-23 and Supplementary Figures 17-19:

These models are based on the following classifier: HGMD and control variants within 1KB of the HGMD variant.
The annotations from Ritchie et al. were used.

Supplementary Table 21. Importance of annotations by Elastic Net

DONOR	1.5258467
CDS	1.48920315
ACCEPTOR	1.36461534
EXON	0.4510464
%GC	0.43019703
SMARCA4	0.31248539
UTR5	0.30175308
GTF2F1	0.23865927
Average.GERP	0.20291391
HDAC2	0.12905712
ENH	0.11773293
STAT2	0.1072002
JUNB	0.08893068
CCNT2	0.05291225
ETS1	0.0489323
TSS	0.04240205
GERP	0.04200631
SRF	0.03347607
SREBF1	0.03091549
PBX3	0.01549044
H3K4me3	0.00772507

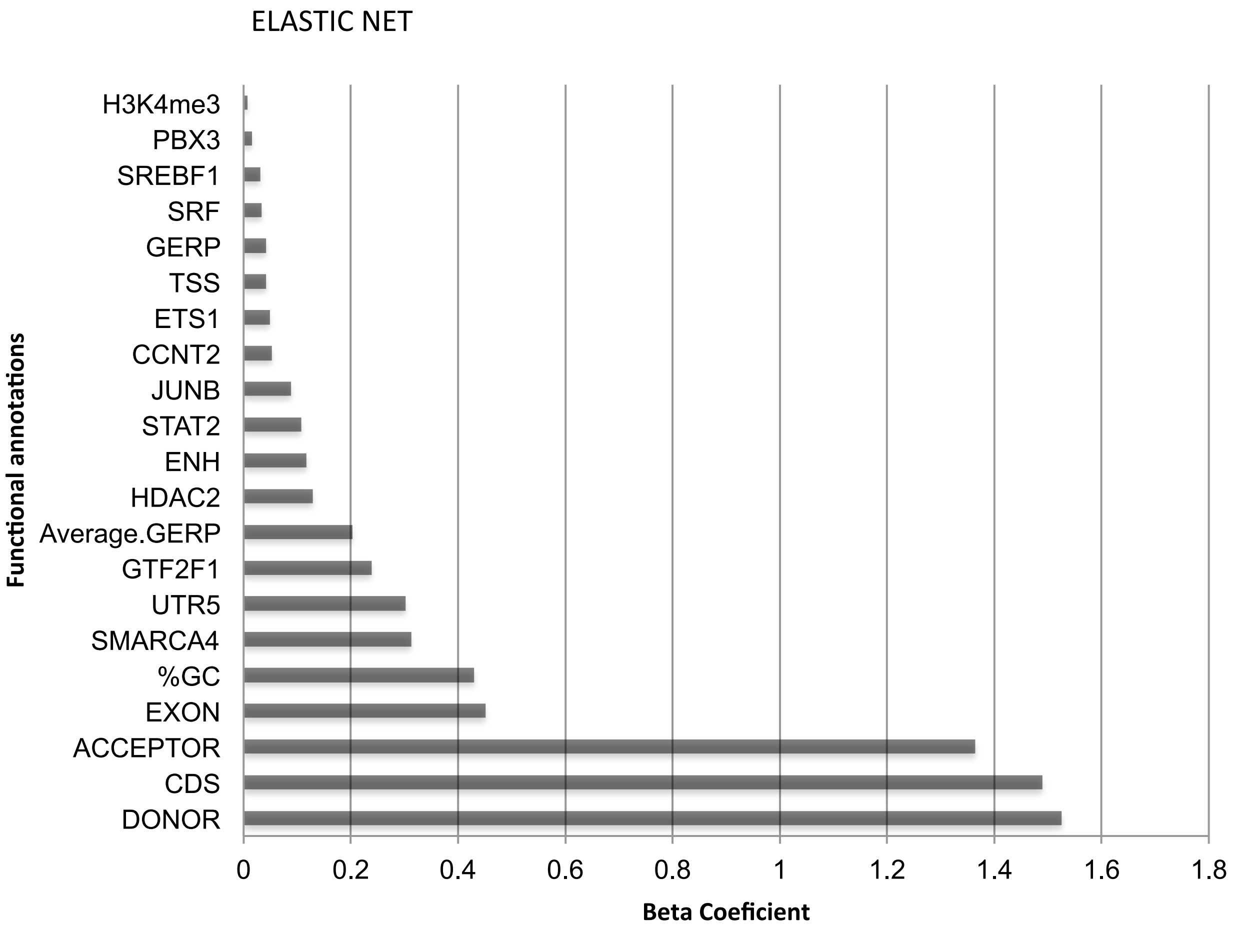

Supplementary Figure 17. Importance of annotations by Elastic Net

Supplementary Table 22. Importance of annotations by Random Forest

RANDOM FOREST --> too many non-zero annots for a graph (134). The top 30 are displayed here.

CDS	0.18943882
EXON	0.16168397
Average.GERP	0.12058937
INTRON	0.08031842
H3K36me3	0.07516592
GERP	0.06150859
H3K4me1	0.03945476
H3K27me3	0.03356697
repeat.	0.03339007
SS.distance	0.03264821
%GC	0.01561323
H3K27ac	0.0133447
DNase	0.01328737
in_cpg	0.01135456
H3K9ac	0.01074734
H3K4me3	0.00938739
H3K4me2	0.00896775
H2AFZ	0.0065741
TSS.distance	0.00619892
H4K20me1	0.00548802
TRAN	0.00541612
UTR5	0.00499614
Average.DAF	0.00497807
UTR3	0.00434988
H3K79me2	0.00434417
Average.het	0.00400752
cpg_island	0.003979
TSS	0.00373025
REP	0.00366999
H3K9me3	0.00281866

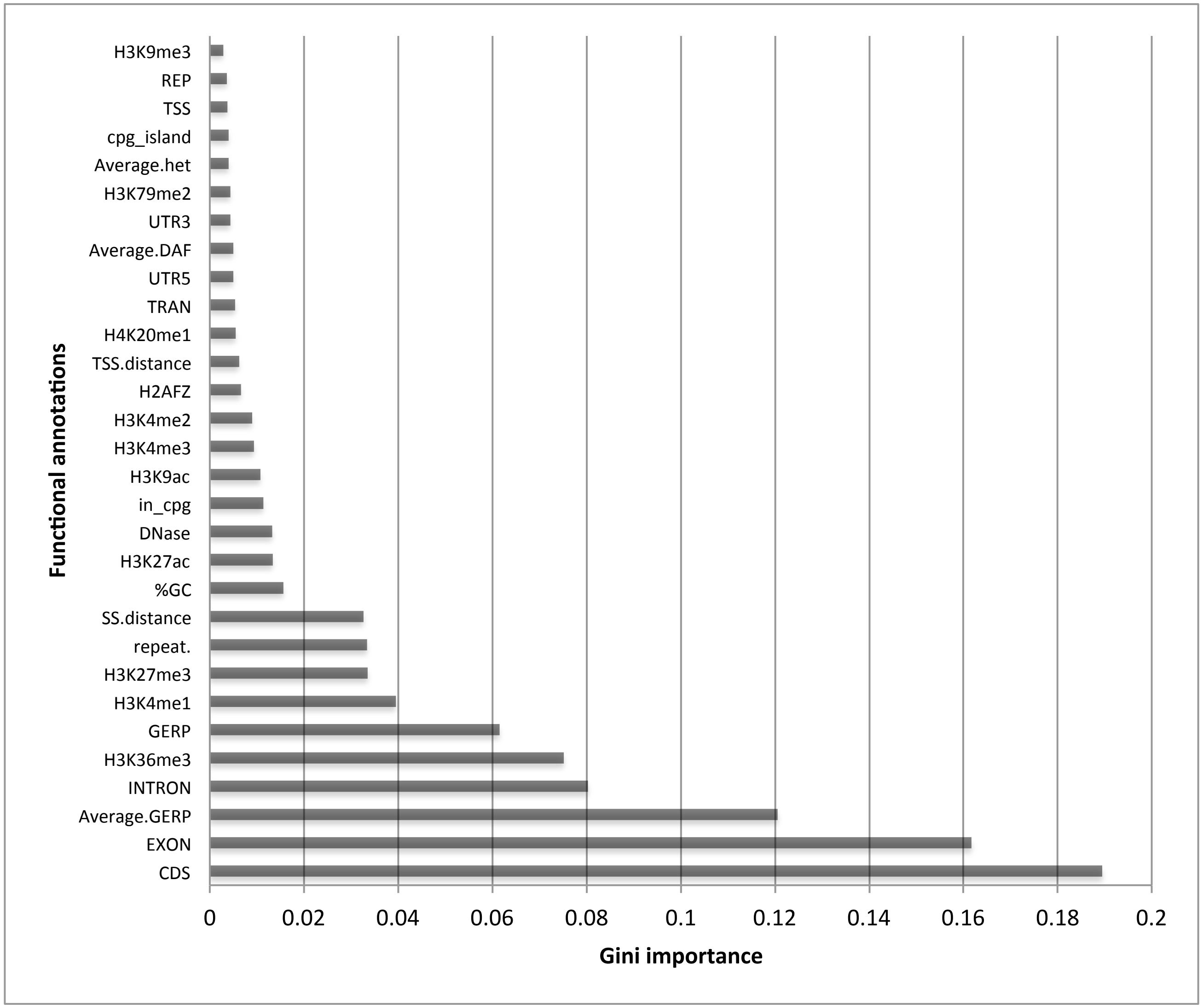

Supplementary Figure 18. Importance of annotations by Random Forest

Supplementary Table 23. Importance of annotations by Support Vector Machine

CDS	0.56149539
EXON	0.41436719
UTR5	0.12996327
DONOR	0.11619774
ACCEPTOR	0.0829269
GTF2F1	0.05462709
JUNB	0.04795783
SRF	0.04726239
PBX3	0.04495562
ETS1	0.0323004
SREBF1	0.02882632
HDAC2	0.02325905
Average.GERP	0.02004804
STAT2	0.01835382
H3K4me3	0.01657522
TSS	0.01337685
ENH	0.01261234
SMARCA4	0.00820819
CCNT2	0.0068465
%GC	0.00449145
GERP	0.00378191

SUPPORT VECTOR MACHINE

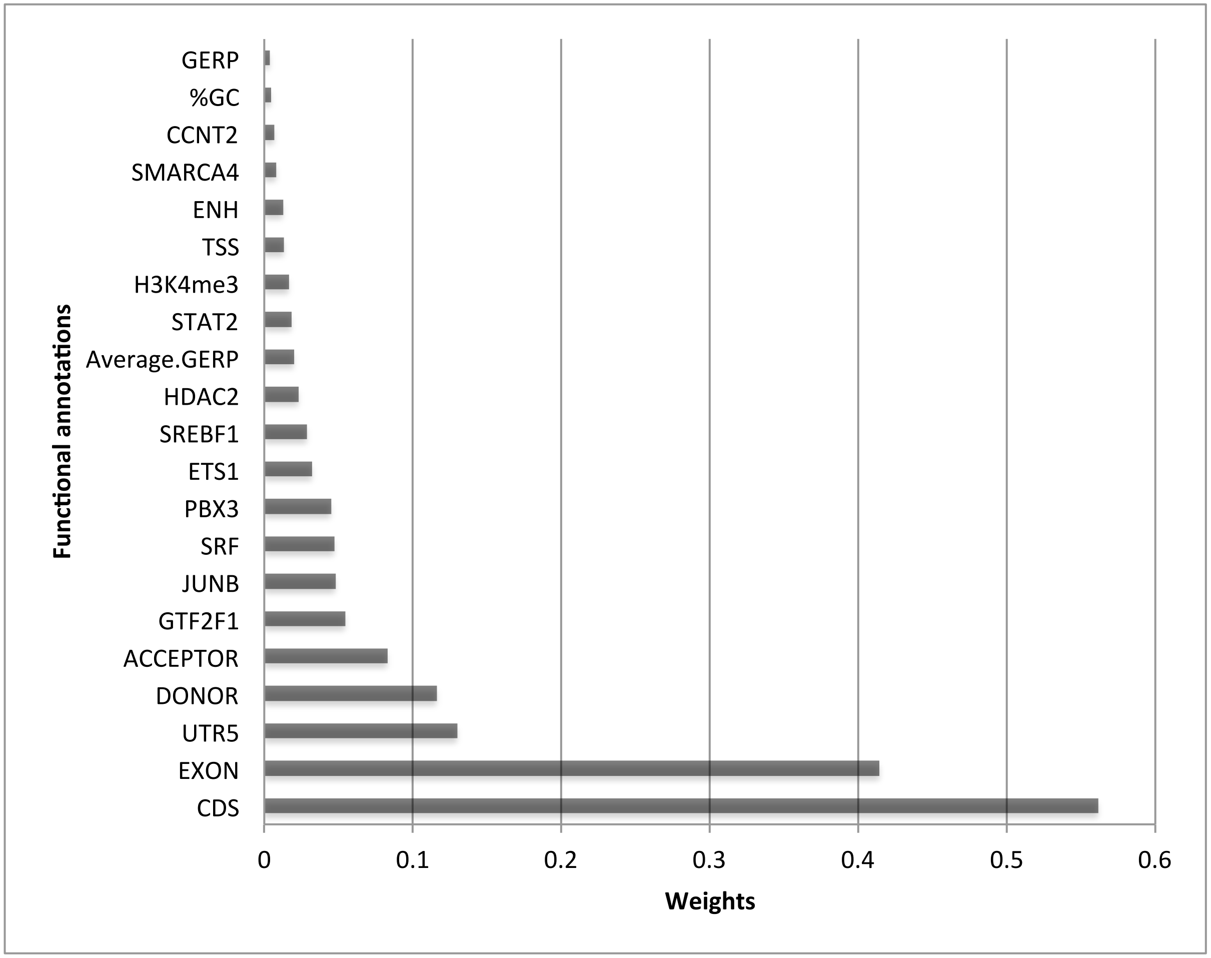

Supplementary Figure 19. Importance of annotations by Support Vector Machine

For Supplementary Tables 21-23 and Supplementary Figures 17-19:

Annotation Legend: See Ritchie et al. for further details.

ACCEPTOR= acceptor splice site
Average.DAF= mean derived allele frequency of variants in 1kb flanking region
Average.GERP= mean GERP score of 100bp flanking region
Average.het= mean heterozygosity of 1kb flanking region
CCNT2= CCNT2 Transcription Factor ChIP-seq peaks
CDS= coding sequence
DNase= DNase1-seq peak
DONOR= donor splice site
ENH= predicted enhancer segment
ETS1= ETS1 Transcription Factor ChIP-seq peaks
EXON= exonic region
GERP= GERP score at the variant locus
GTF2F1= GTF2F1 Transcription Factor ChiP-seq peaks
H2AFZ= H2AFZ Histone modification ChIP-seq peaks
H3K27ac= H3K27ac Histone modification ChIP-seq peaks
H3K27me3= H3K27me3 Histone modification ChIP-seq peaks
H3K36me3= H3K36me3 Histone modification ChIP-seq peaks
H3K4me1= H3K4me1 Histone modification ChIP-seq peaks
H3K4me2= H3K4me2 Histone modification ChIP-seq peaks
H3K4me3= H3K4me3 Histone modification ChIP-seq peaks
H3K79me2= H3K79me2 Histone modification ChIP-seq peaks
H3K9ac= H3K9ac Histone modification ChIP-seq peaks
H3K9me3= H3K9me3 Histone modification ChIP-seq peaks
H4K20me1= H4K20me1 Histone modification ChIP-seq peaks
HDAC2= HDAC2 Transcription Factor ChIP-seq peaks
INTRON= intronic region
JUNB= JUNB Transcription Factor ChIP-seq peaks
PBX3= PBX3 Transcription Factor ChIP-seq peaks
REP= predicted repressed sequence

SMARCA4= SMARCA4 Transcription Factor ChIP-seq peaks
SREBF1= SREBF1 Transcription Factor ChIP-seq peaks
SRF= SRF Transcription Factor ChIP-seq peaks
SS.distance= distance to the nearest splice site
STAT2= STAT2 Transcription Factor ChIP-seq peaks
TRAN= predicted transcribed segment
TSS= predicted promoter segment
TSS.distance= distance to the nearest TSS
UTR3= 3 prime UTR
UTR5= 5 prime UTR
%GC= GC content of 100bp flanking region
cpg_island= Predicted CpG island
in_cpg= reference sequence at variant locus is a CpG dinucleotide
repeat.= annotated repeat element

Supplementary Tables 24-26 and Supplementary Figures 20-22:

These models are based on the following classifier: HGMD and control variants within 1KB of the HGMD variant.

The annotations from Kircher et al. were used.

Supplementary Table 24. Importance of annotations by Elastic Net

CpG	1.64257081
YxY	0.61902895
U5xpriPhCons	0.61425523
ConsequencexSG	0.54174906
nAAx*	0.43276376
SIFTval	0.16734979
verPhCons	0.14840771
GerpN	0.14715183
ConsequencexNS	0.14189243
priPhCons	0.13669347
PolyPhenCatxbenign	0.13499441
NSxminDistTSS	0.11727677
CSxminDistTSS	0.10941404
verPhyloP	0.04275962
minDistTSE	0.0403301
SGxminDistTSS	0.03900904
SxminDistTSS	0.0192986
TFBS	0.0111528
NCxminDistTSS	0.00937678
SegwayxTF0	0.00856464
NSxminDistTSE	0.00389292
KxK	0.0024366
SGxbStatistic	0.00050175
EncExp	0.00023775
TFBSPeaksMax	0.00014412

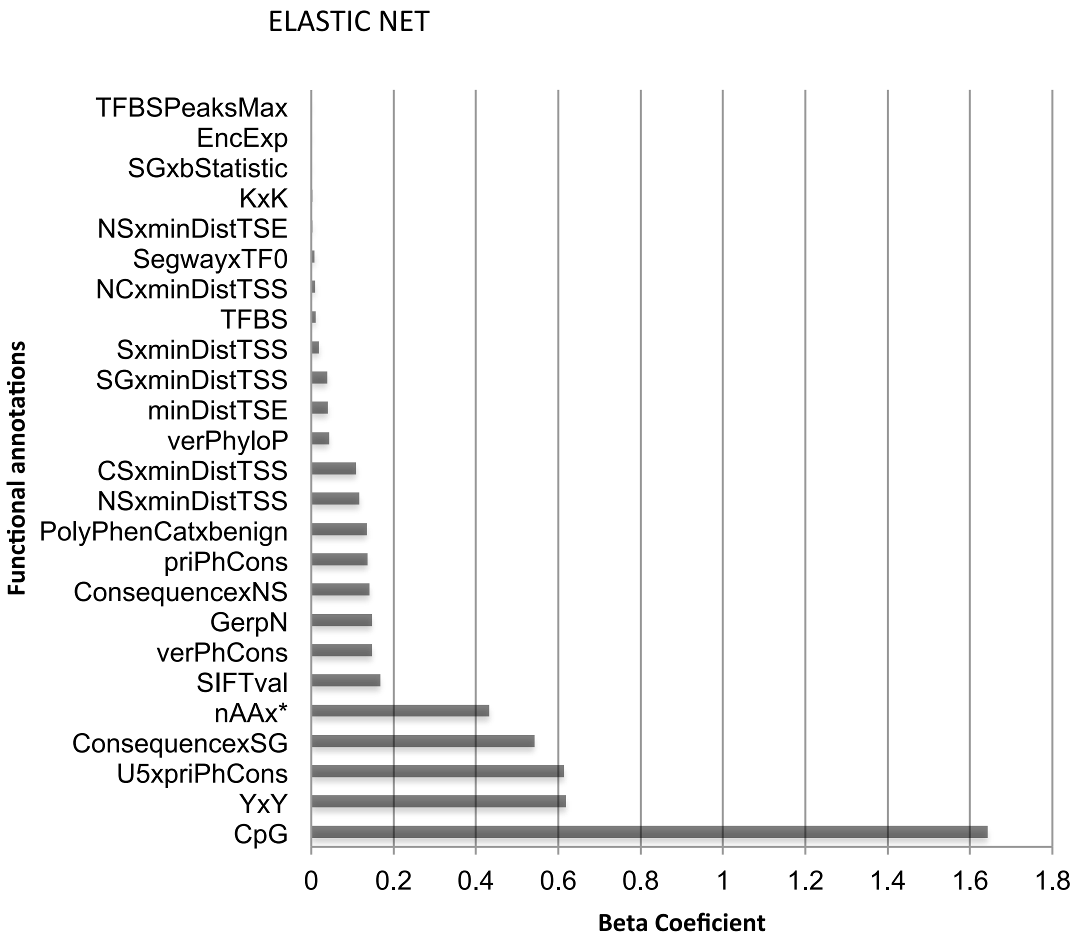

Supplementary Figure 20. Importance of annotations by Elastic Net

Supplementary Table 25. Importance of annotations by Random Forest

protpos	0.04590905
NSxCDSpos	0.04081936
IND_PolyPhenVal	0.04077479
IND_protpos	0.03701
PolyPhenCatxUD	0.0358595
NSxrelCDSpos	0.03321642
CDSpos	0.03199467
NSxcDNApos	0.03029051
relcDNApos	0.02894276
IND_relCDSpos	0.02860594
NSxGerpN	0.02815068
IND_Grantham	0.02462413
PolyPhenVal	0.02247068
SIFTcatxUD	0.02148823
IxpriPhCons	0.01823818
NSxbStatistic	0.01731354
GerpRS	0.0172512
NSxminDistTSE	0.01699031
GerpRSpval	0.01624278
NSxminDistTSS	0.0160178
IND_relcDNApos	0.0152251
nAAxUD	0.01447571
NSxprotpos	0.0144745
IxbStatistic	0.01376113
NSxGerpS	0.01180528
NSxverPhyloP	0.01163979
NSxrelcDNApos	0.01059885
SIFTval	0.01045299
mamPhCons	0.01013812
IxGerpN	0.00981919

RANDOM FOREST --> too many non-zero annots for a graph (430).
The top 30 are displayed here.

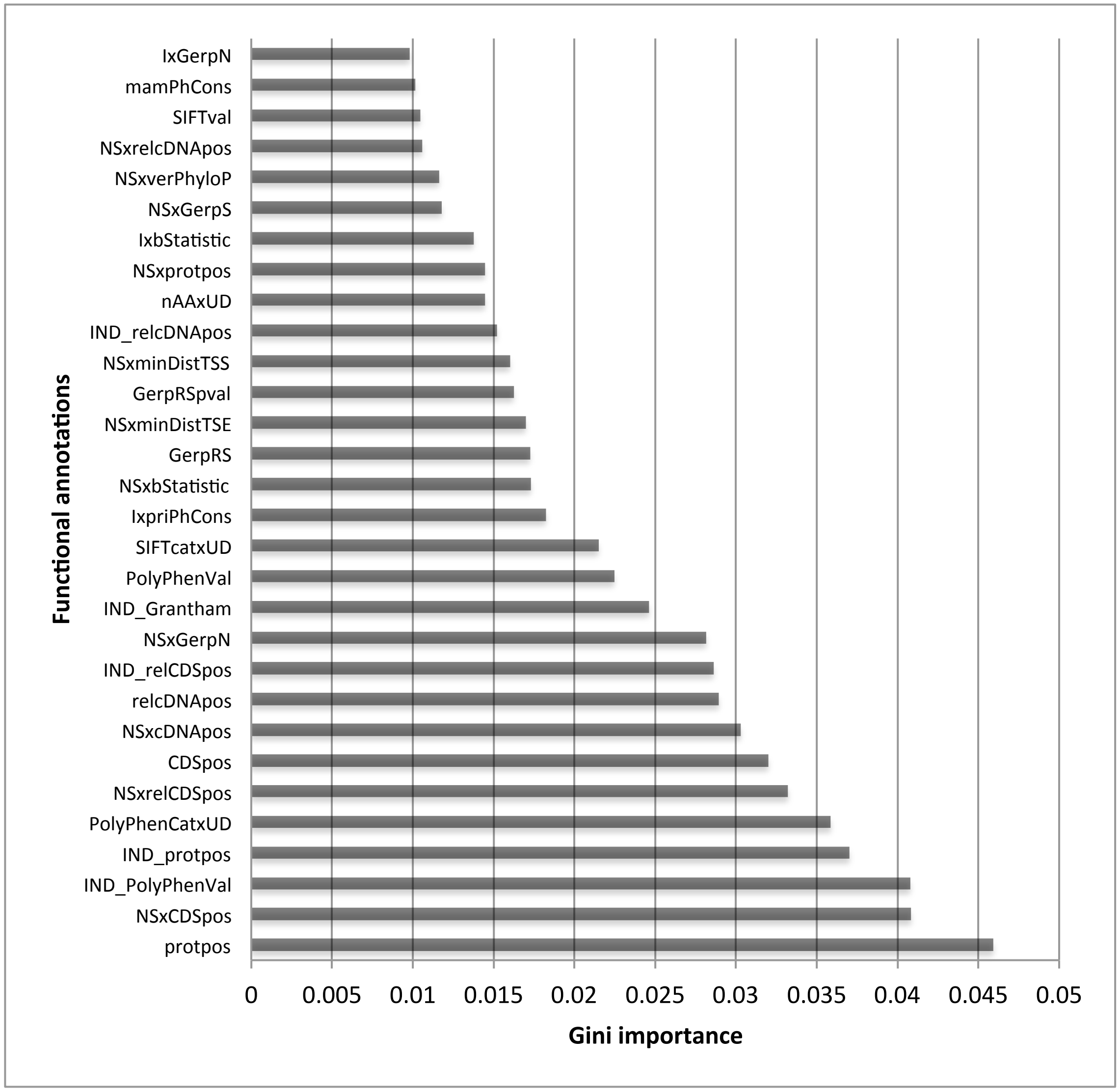

Supplementary Figure 21. Importance of annotations by Random Forest

Supplementary Table 26. Importance of annotations by Support Vector Machine

ConsequencexNS	0.34914625
NSxminDistTSE	0.213846
NSxminDistTSS	0.20693293
TFBS	0.1665795
SxminDistTSS	0.12067178
CSxminDistTSS	0.11274411
PolyPhenCatxbenign	0.10430502
YxY	0.10426699
verPhCons	0.09479932
NCxminDistTSS	0.09210029
CpG	0.08386596
ConsequencexSG	0.06312334
nAAx*	0.06312334
verPhyloP	0.06262857
SIFTval	0.06106331
GerpN	0.0555322
priPhCons	0.05166973
U5xpriPhCons	0.05113698
EncExp	0.04900535
SGxbStatistic	0.04323714
SGxminDistTSS	0.04206416
TFBSPeaksMax	0.04194938
KxK	0.03740145
SegwayxTF0	0.01519715
minDistTSE	-0.0160579

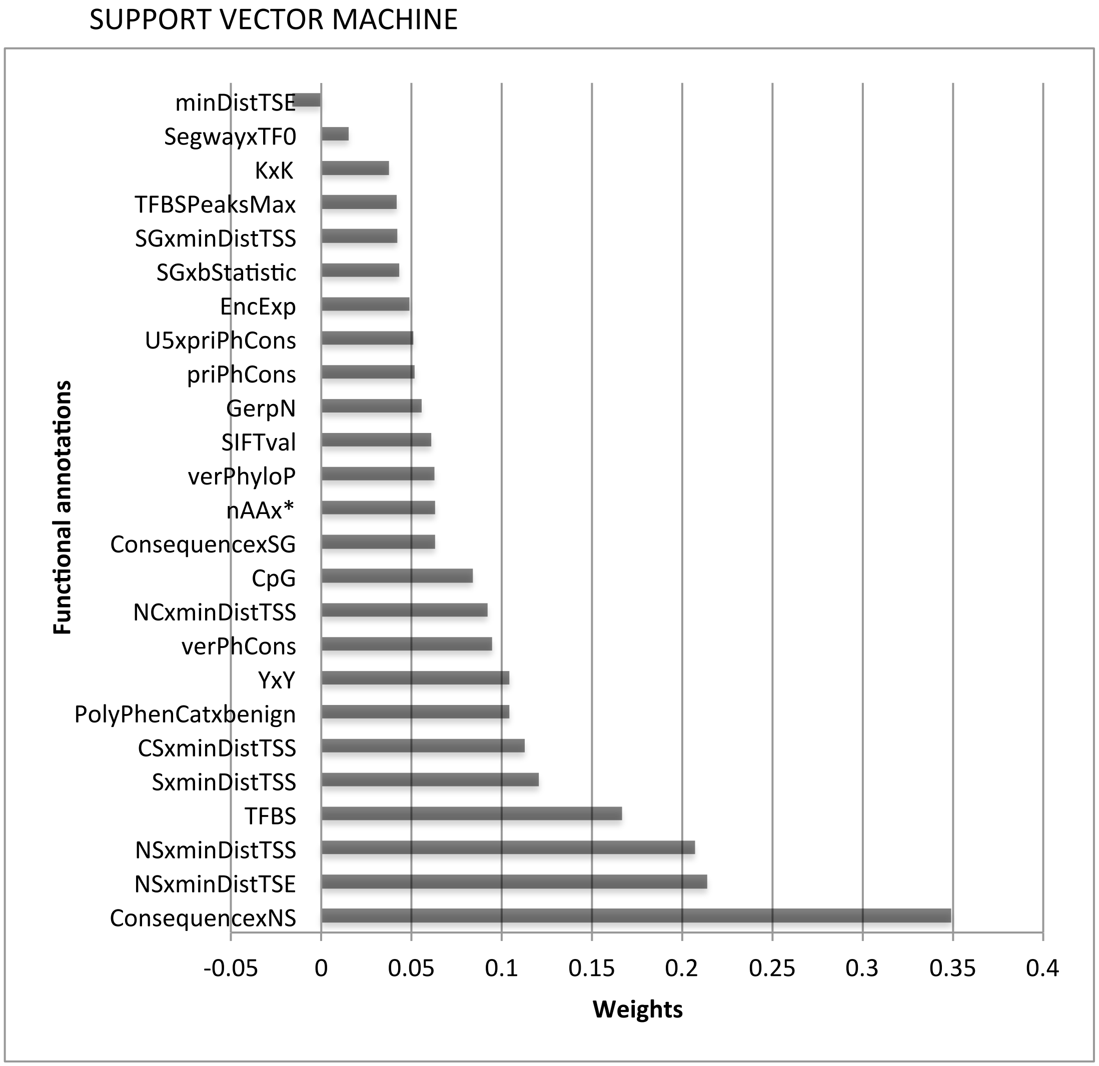

Supplementary Figure 22. Importance of annotations by Support Vector Machine

For Supplementary Tables 24-26 and Supplementary Figures 20-22:

Annotation Legend: See Kircher et al. for further details.

CDSpos= Base position from coding start
CSxminDistTSS= interaction between canonical splice and Distance to closest Transcribed Sequence Start (TSS)
ConsequencexNS= nonsynonymous
ConsequencexSG= stop-gained
CpG= Percent CpG in a window of +/- 75bp
EncExp= Maximum ENCODE expression value
GerpN= Neutral evolution score defined by GERP++
GerpRS= Gerp element score
GerpRSpval= Gerp element p-Value
IND_Grantham= indicator variable for Grantham score: oAA,nAA
IND_PolyPhenVal= PolyPhen score
IND_protpos= indicator variable for Amino acid position from coding start
IND_relCDSpos= indicator variable for Relative position in coding sequence
IND_relcDNApos= indicator variable for Relative position in transcript
IxGerpN= interaction between intronic and Neutral evolution score defined by GERP++
IxbStatistic= interaction between intronic and Background selection score
IxpriPhCons= interaction between intronic and Primate PhastCons conservation score (excl. human)
KxK= interaction between previous amino acid lysine and new amino acid lysine
NCxminDistTSS= interaction between noncoding and Distance to closest Transcribed Sequence Start (TSS)
NSxCDSpos= interaction between nonsynonymous and Base position from transcription start
NSxGerpN= interaction between nonsynonymous and Neutral evolution score defined by GERP++
NSxGerpS= interaction between nonsynonymous and Rejected Substitution' score defined by GERP++
NSxbStatistic= interaction between nonsynonymous and Background selection score
NSxcDNApos= interaction between nonsynonymous and Base position from transcription start
NSxminDistTSE= interaction between nonsynonymous and Distance to closest Transcribed Sequence End (TSE)
NSxminDistTSS= interaction between nonsynonymous and Distance to closest Transcribed Sequence Start (TSS)
Nsxprotpos= interaction between nonsynoymous and Amino acid position from coding start
NSxrelCDSpos= interaction between nonsynonymous and Relative position in coding sequence
NSxrelcDNApos= interaction between nonsynonymous and Relative position in transcript

NSxverPhyloP= interaction between nonsynonymous and Vertebrate PhyloP (excl. human)
PolyPhenCatxUD= PolyPhen category, undefined
PolyPhenCatxbenign= PolyPhen category, benign
PolyPhenVal= PolyPhen scorel
SGxbStatistic= interaction between stop-gained and Background selection score
SGxminDistTSS= interaction between stop-gained and Distance to closest Transcribed Sequence Start (TSS)
SIFTcatxUD= SIFT category, undefined
SIFTval= SIFT score
SegwayxTF0= Segway, TFO category
SxminDistTSS= interaction between synonymous and Distance to closest Transcribed Sequence Start (TSS)
TFBS= Number of different overlapping ChIP transcription factor binding sites
TFBSPeaksMax= Maximum value of overlapping ChIP transcription factor binding site peaks across cell types/tissue
U5xpriPhCons= interaction between 5Prime UTR and Primate PhastCons conservation score (excl. human)

Supplementary Tables 27-29 and Supplementary Figures 23-25:

These models are based on the following classifier: non-exonic HGMD and non-exonic control variants within 1KB of the HGMD variant. The annotations from Gagliano et al. were used.

Supplementary Table 27. Importance of annotations by Elastic Net

DNase_I	0.91216267
GTEx_eQTLs	0.34362132
UK_Brain_eQTLs	0.26032036
H3K4Me1	0.17681986
PhyloP	0.00330053
PhastCons	0.00195327
splice	0
nonsynonymous	0
TFBS	0
miRNA	0
Gencode_Txnstart	0
H3K27Ac	-0.2236143
H3K4Me3	-0.2411067
UCSC_Genes	-0.2931357

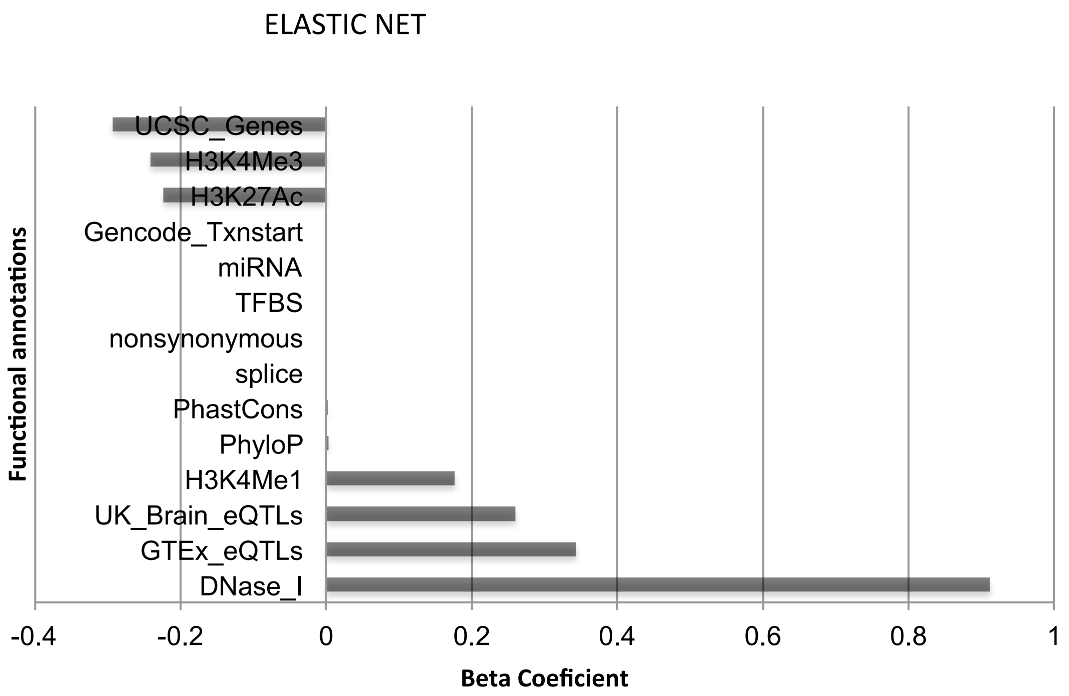

Supplementary Figure 23. Importance of annotations by Elastic Net

Supplementary Table 28. Importance of annotations by Random Forest

DNase_I	0.31980572
PhastCons	0.20783459
PhyloP	0.16117035
H3K4Me1	0.08249287
UCSC_Genes	0.07202067
UK_Brain_eQTLs	0.04456005
TFBS	0.03965993
H3K27Ac	0.03478116
H3K4Me3	0.02484832
GTEx_eQTLs	0.01158394
nonsynonymous	0.00111109
Gencode_Txnstart	0.00013131
miRNA	0
splice	0

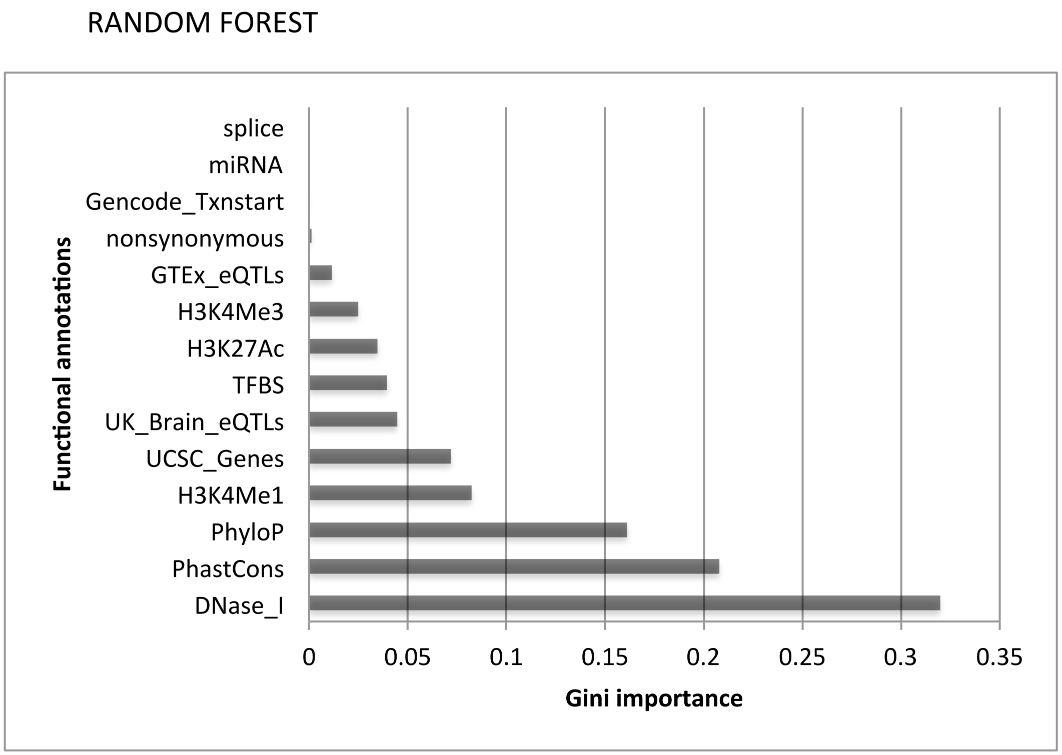

Supplementary Figure 24. Importance of annotations by Random Forest

Supplementary Table 29. Importance of annotations by Support Vector Machine

SUPPORT VECTOR MACHINE

DNase_I	0.55783189
UK_Brain_eQTLs	0.18788467
H3K4Me1	0.17503961
H3K27Ac	0.14812591
H3K4Me3	0.09582921
PhyloP	0.09328432
PhastCons	0.08604586
GTEx_eQTLs	0.08190499
UCSC_Genes	-0.0003697

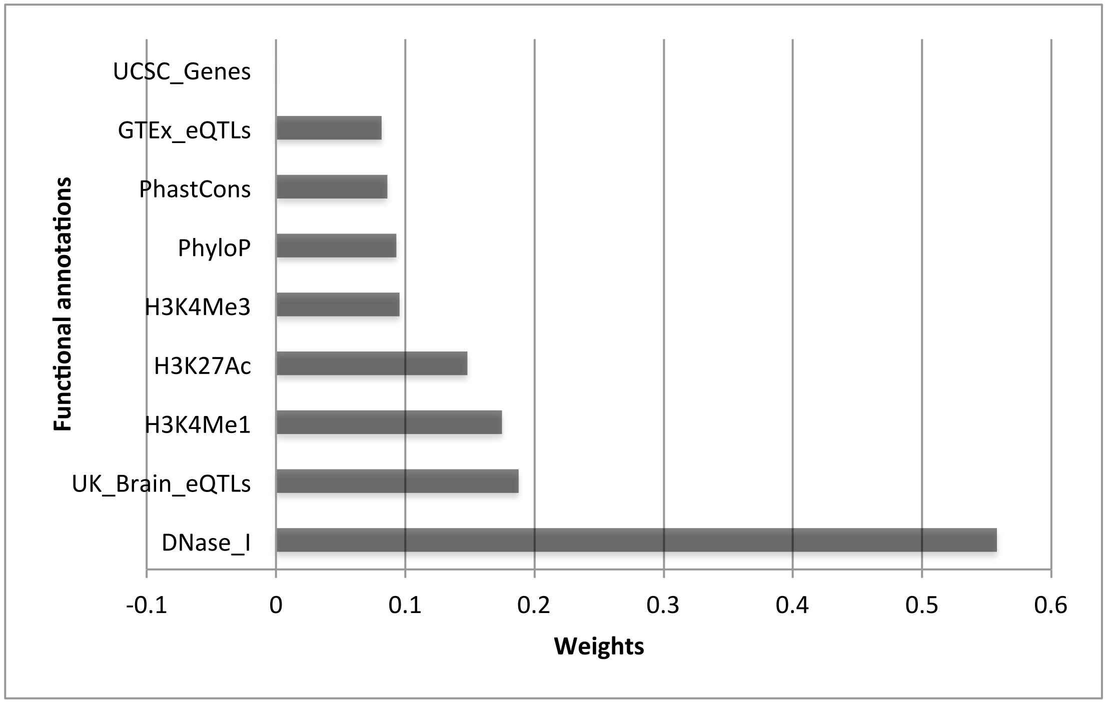

Supplementary Figure 25. Importance of annotations by Support Vector Machine

For Supplementary Tables 27-29 and Supplementary Figures 23-25:

Annotation Legend: See Gagliano et al. for further details.

Nonsynonymous= Nonsynonymous SNP
GTEx_eQTLs= cis eQTL from the GTEx Project
TFBS= Transcription factor binding site
DNase_I= DNaseI hypersensitive site
H3K4Me3= H4K4Me3 histone modification
UCSC_Genes= UCSC Gene
UK_Brain_eQTLs= cis eQTL from the UK Brain Consortium
H3K27Ac= H3K27Ac histone modification
PhyloP= PhyloP conservation score
Splice= +/-5 base pairs from a splice site
PhastCons= PhastCons conservation score
H3K4Me1= H3K4Me1 histone modification
miRNA= microRNA target as defined by TargetScan
Gencode_Txnstart= Transcription start site as defined by Gencode

Supplementary Tables 30-32 and Supplementary Figures 26-28:

These models are based on the following classifier: non-exonic HGMD and non-exonic control variants within 1KB of the HGMD variant. The annotations from Ritchie et al. were used.

Supplementary Table 30. Importance of annotations by Elastic Net

ACCEPTOR	1.328016
DONOR	1.13231198
%GC	0.77416304
Average.GERP	0.42610847
HDAC2	0.31861927
PBX3	0.15134738
H3K4me2	0.05150363
ENH	0.04571758
ELF1	0.04362891
H2AFZ	0.0286725
H3K27me3	0.01442794
H3K4me3	0.01127367
SS.distance	7.46E-07

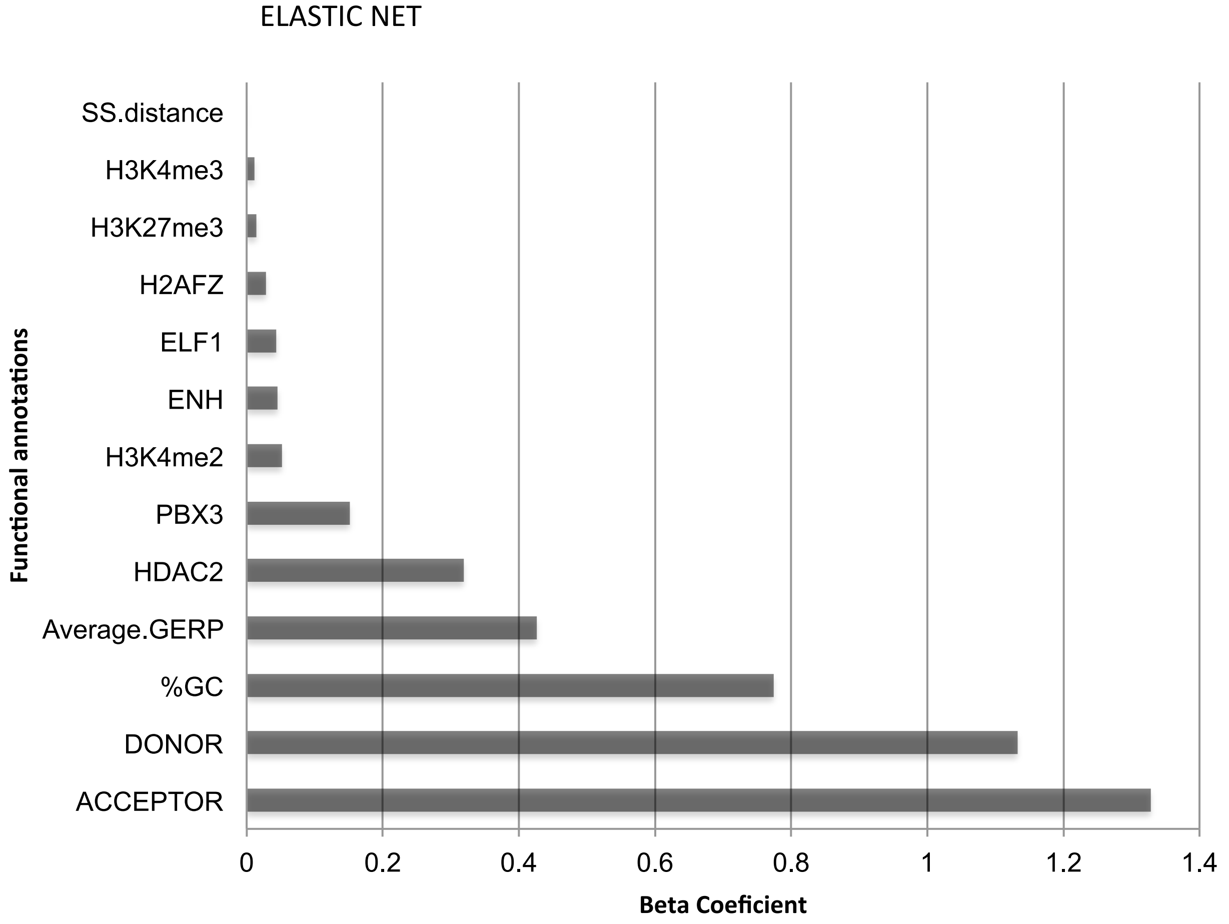

Supplementary Figure 26. Importance of annotations by Elastic Net

Supplementary Table 31. Importance of annotations by Random Forest

RANDOM FOREST --> too many non-zero annots for a graph (124). The top 30 are displayed here

Average.GERP	0.10839647
TSS.distance	0.10727704
SS.distance	0.06808475
INTRON	0.0601164
GERP	0.05273117
H3K36me3	0.04625738
H3K4me3	0.03874786
DNase	0.03345831
dnase_fps	0.03257359
repeat.	0.03186024
Average.het	0.03112989
H3K4me2	0.0310369
H3K27me3	0.03055748
Average.DAF	0.03039181
TRAN	0.02670498
%GC	0.02655676
H2AFZ	0.02403689
FAIRE	0.01643756
H3K4me1	0.01545784
TSS	0.01528586
DONOR	0.01467283
H3K27ac	0.01451522
ENH	0.01331986
H3K9ac	0.0103722
H4K20me1	0.00982251
REP	0.00941113
H3K79me2	0.00756666
bound_motifs	0.00549367
POLR2A	0.00542883
cpg_island	0.00542017

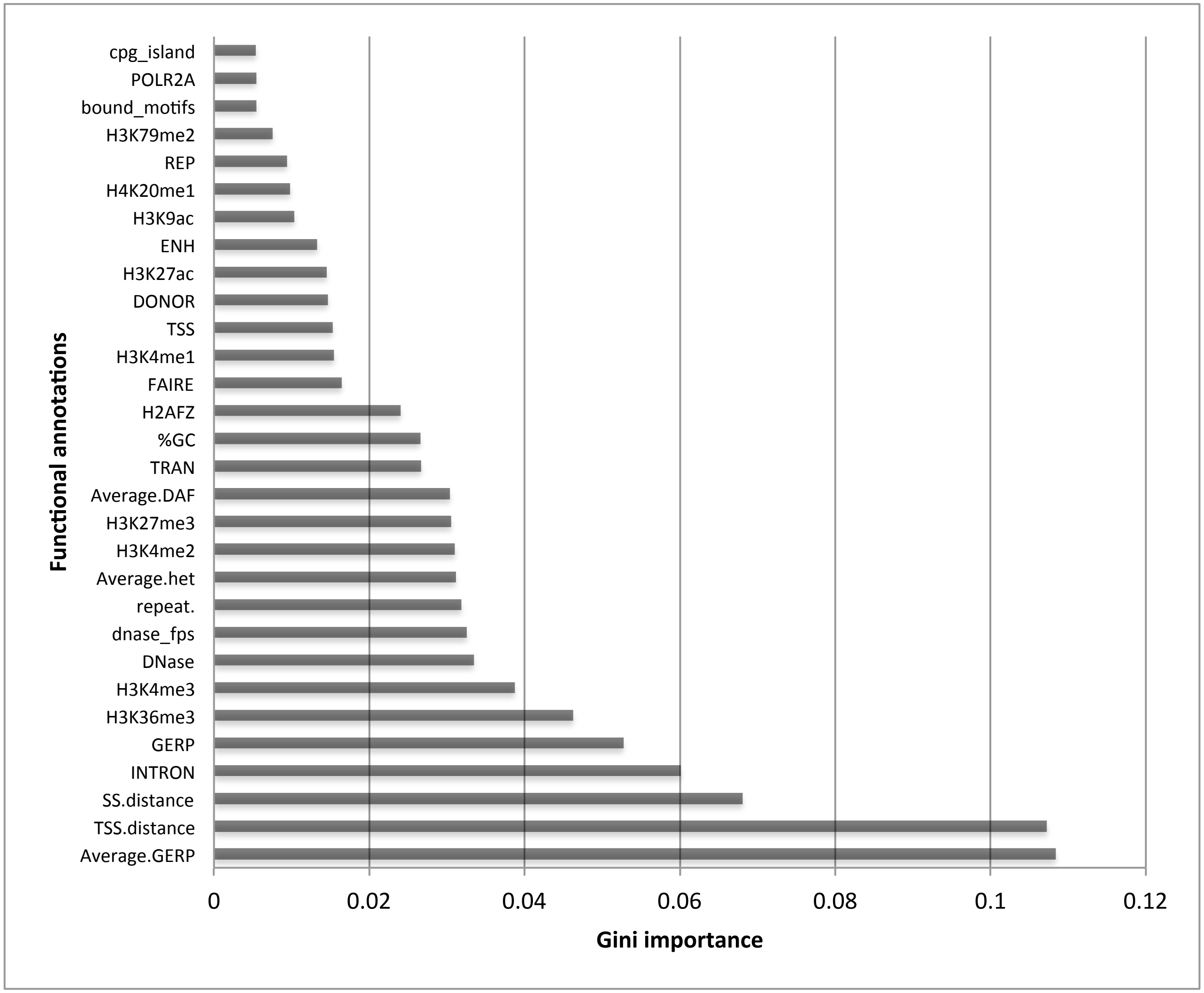

Supplementary Figure 27. Importance of annotations by Random Forest

Supplementary Table 32. Importance of annotations by Support Vector Machine

H3K27me3	0.23132127
Average.GERP	0.21730765
H3K4me2	0.21324897
DONOR	0.16474043
H3K4me3	0.16167312
H2AFZ	0.15065681
ENH	0.14814975
ACCEPTOR	0.12629488
%GC	0.0945848
HDAC2	0.07381445
ELF1	0.06236036
SS.distance	0.04800924
PBX3	0.03475798

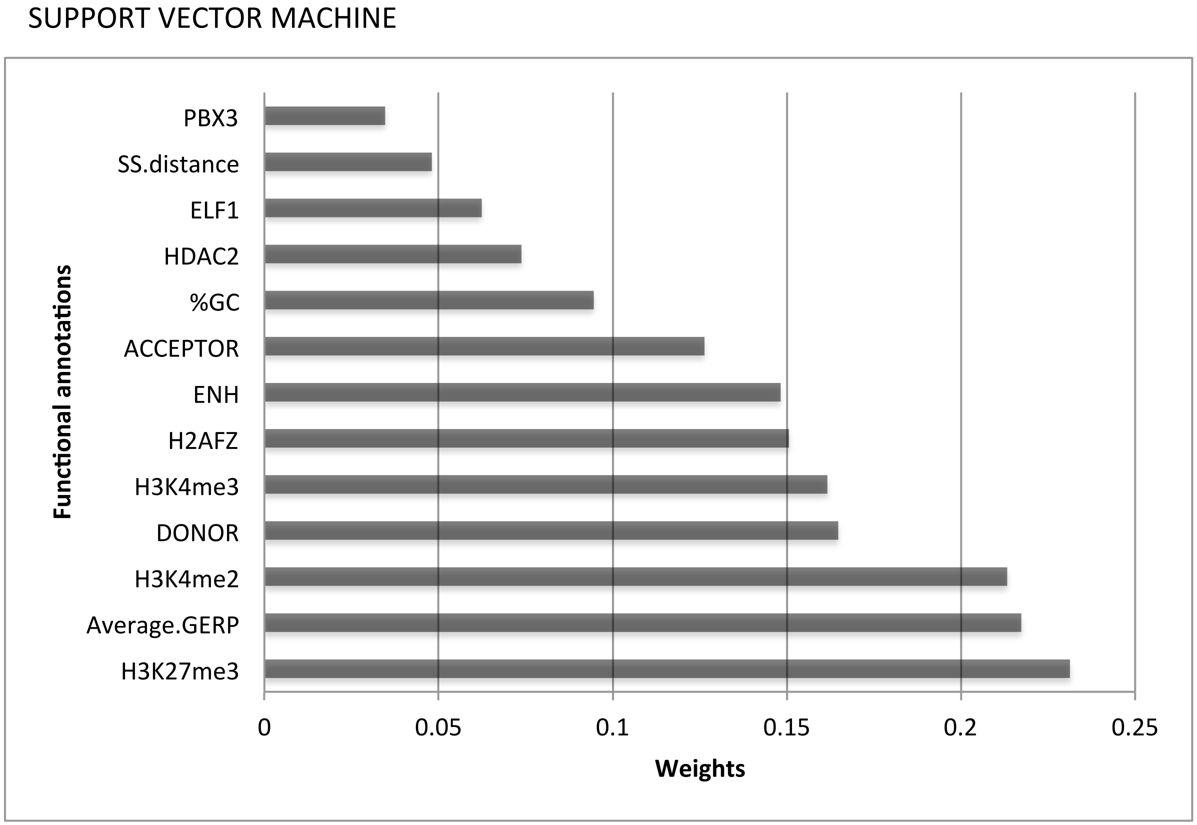

Supplementary Figure 28. Importance of annotations by Support Vector Machine

For Supplementary Tables 30-32 and Supplementary Figures 26-28:

Annotation Legend: See Ritchie et al. for further details.

ACCEPTOR= acceptor splice site
Average.DAF= mean derived allele frequency of variants in 1kb flanking region
Average.GERP= mean GERP score of 100bp flanking region
Average.het= mean heterozygosity of 1kb flanking region
DNase= DNase1-seq peak
DONOR= donor splice site
ELF1= ELF1 Transcription Factor ChIP-seq peaks
ENH= predicted enhancer segment
FAIRE= FAIRE-seq peak
GERP= GERP score at the variant locus
H2AFZ= H2AFZ Histone modification ChIP-seq peaks
H3K27ac= H3K27ac Histone modification ChIP-seq peaks
H3K27me3= H3K27me3 Histone modification ChIP-seq peaks
H3K36me3= H3K36me3 Histone modification ChIP-seq peaks
H3K4me1= H3K4me1 Histone modification ChIP-seq peaks
H3K4me2= H3K4me2 Histone modification ChIP-seq peaks
H3K4me3= H3K4me4 Histone modification ChIP-seq peaks
H3K79me2= H3K79me2 Histone modification ChIP-seq peaks
H3K9ac= H3K9ac Histone modification ChIP-seq peaks
H4K20me1= H3K20me1 Histone modification ChIP-seq peaks
HDAC2= HDAC2 Transcription Factor ChIP-seq peaks
INTRON= intronic region
PBX3= PBX3 Transcription Factor ChIP-seq peaks
POLR2A= POLR2A Transcription Factor ChIP-seq peaks
REP= predicted repressed sequence
SS.distance= distance to the nearest splice site
TRAN= predicted transcribed segment
TSS= predicted promoter segment
TSS.distance= distance to the nearest TSS

%GC= GC content of 100bp flanking region
bound_motifs= bound transcription factor motifs
cpg_island= Predicted CpG island
dnase_fps= DNase1-seq footprint
repeat.= annotated repeat element

Supplementary Tables 33-35 and Supplementary Figures 29-31:

These models are based on the following classifier: non-exonic HGMD and non-exonic control variants within 1KB of the HGMD variant. The annotations from Kircher et al. were used.

Supplementary Table 33. Importance of annotations by Elastic Net

EncOCFaireSig	2.00275077
EncOCpolIISig	0.60623754
Dst2SplTypexDONOR	0.13618017
DNxpriPhyloP	0.13403555
SegwayxL1	0.11259132
ConsequencexCS	0.1105286
GerpN	0.1065717
minDistTSE	0.09535823
AltxG	0.09345319
priPhCons	0.09003158
mamPhCons	0.08869554
Dst2SplTypexACCEPTOR	0.06263028
SegwayxC0	0.06199754
ConsequencexUP	0.06007579
SegwayxF0	0.04442006
UPxGerpN	0.0397594
SegwayxTF0	0.03347192
CxG	0.0258295
CSxminDistTSS	0.01964801
UPxminDistTSE	0.01457942
EncOCFairePVal	0.01010597
EncOCCombPVal	0.00856037
TFBS	0.00690565
EncOCDNasePVal	0.00642198
UPxminDistTSS	0.00577863
EncOCctcfPVal	0.0053397
EncH3K4Me1	0.00402159

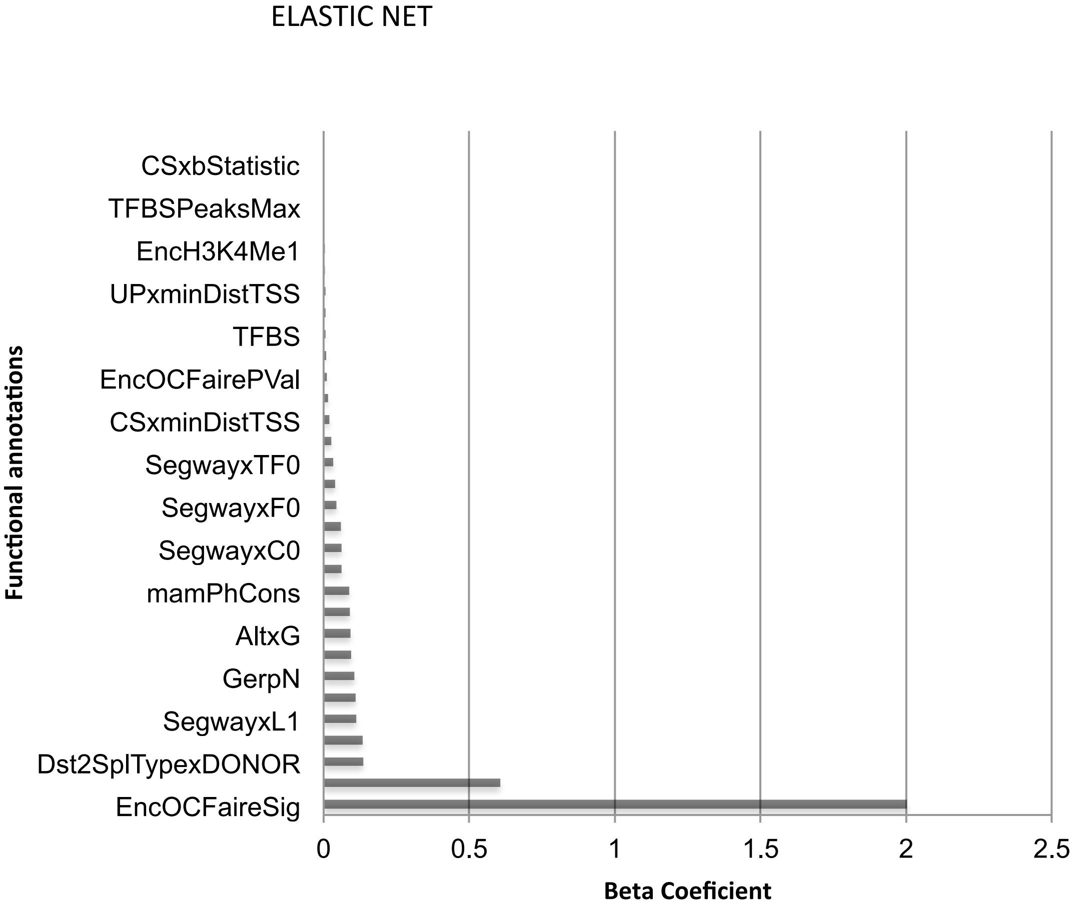

Supplementary Figure 29. Importance of annotations by Elastic Net

EncExp	0.00118442
TFBSPeaksMax	0.00062729
TFBSPeaks	0.00024418
CSxbStatistic	0.00014575
UPxbStatistic	0.00011621

Supplementary Table 34. Importance of annotations by Random Forest

IxminDistTSS	0.03505276
Consequencexl	0.03298746
GerpN	0.03114855
minDistTSS	0.02957615
IxminDistTSE	0.02664504
IxGerpN	0.02660732
minDistTSE	0.02567448
IxpriPhCons	0.02146826
TFBSPeaksMax	0.01816294
UPxminDistTSE	0.01772384
EncH3K4Me1	0.01672854
GerpRS	0.01622944
GerpRSpval	0.01592789
mamPhCons	0.01510546
EncH3K4Me3	0.01470354
Dst2SplTypexUD	0.01424108
IxmamPhCons	0.01386881
verPhCons	0.01378669
RxminDistTSS	0.01374052
EncOCctcfSig	0.01362472
verPhyloP	0.01343313
IxbStatistic	0.01299017
Dst2Splice	0.01282226
UPxGerpN	0.01271164
EncOCpolIIPVal	0.01256621
priPhyloP	0.01252237
IxverPhyloP	0.01195523
EncOCDNasePVal	0.01193051
bStatistic	0.01172126
GC	0.01144353

RANDOM FOREST

--> too many non-zero annots for a graph (175). The top 30 are displayed here.

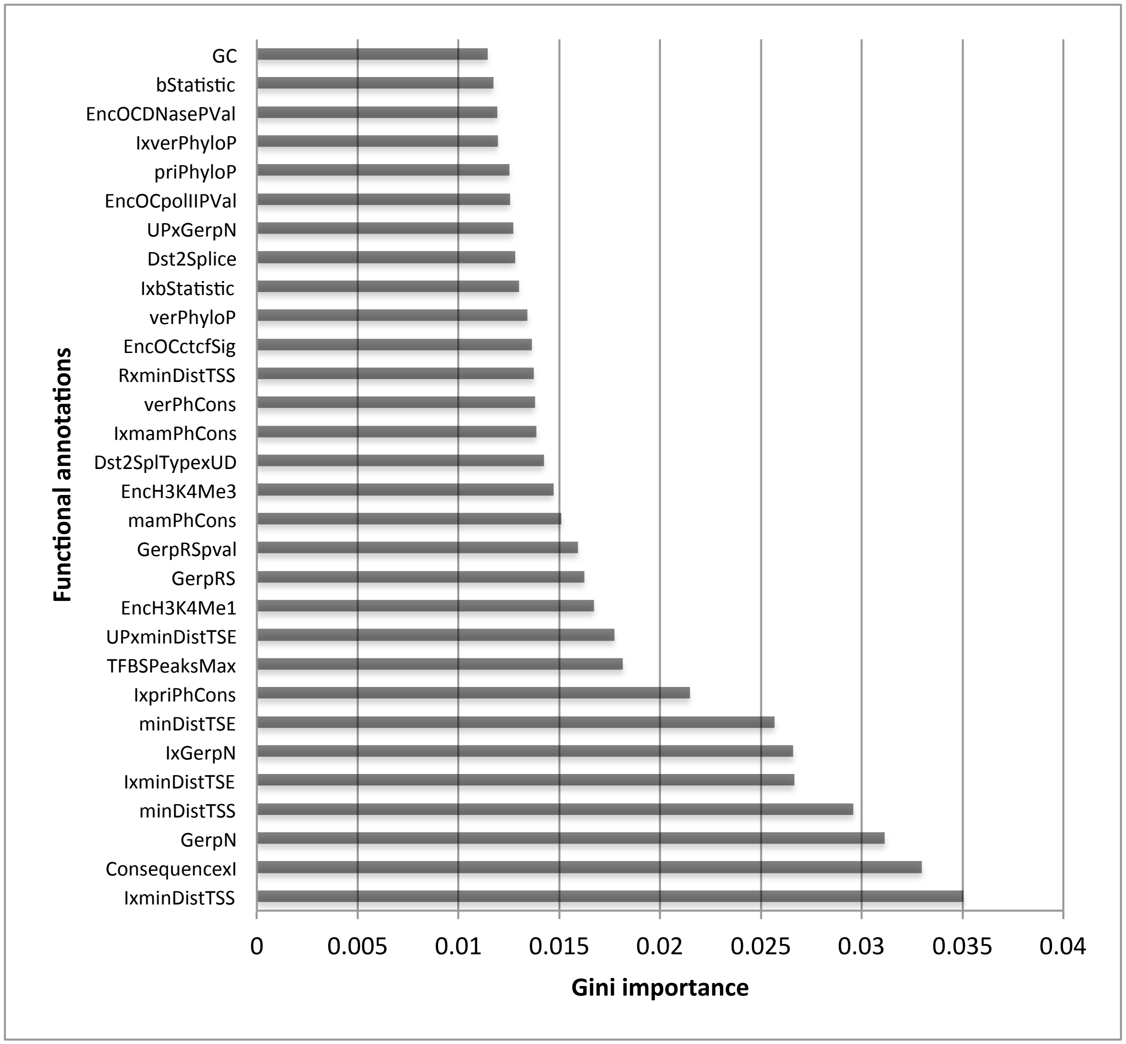

Supplementary Figure 30. Importance of annotations by Random Forest

Supplementary Table 35. Importance of annotations by Support Vector Machine

UPxbStatistic	0.43664711
TFBSPeaksMax	0.32498967
EncOCctcfPVal	0.30378731
Dst2SplTypexACCEPTOR	0.29170818
mamPhCons	0.26782301
SegwayxTF0	0.2649652
EncOCDNasePVal	0.21291776
ConsequencexUP	0.18995155
UPxminDistTSE	0.18435745
EncExp	0.1832309
EncOCpolIISig	0.18029964
Dst2SplTypexDONOR	0.17574117
CxG	0.14940578
EncOCCombPVal	0.14830761
EncOCFaireSig	0.12933384
SegwayxC0	0.09815048
EncH3K4Me1	0.07896724
CSxminDistTSS	0.07502759
CSxbStatistic	0.05672659
GerpN	0.04950504
TFBS	0.04091979
minDistTSE	0.03162832
DNxpriPhyloP	0.00788203
priPhCons	0.0078501
SegwayxL1	0.0047192
AltxG	-0.0067476
SegwayxF0	-0.0078445
ConsequencexCS	-0.0189846

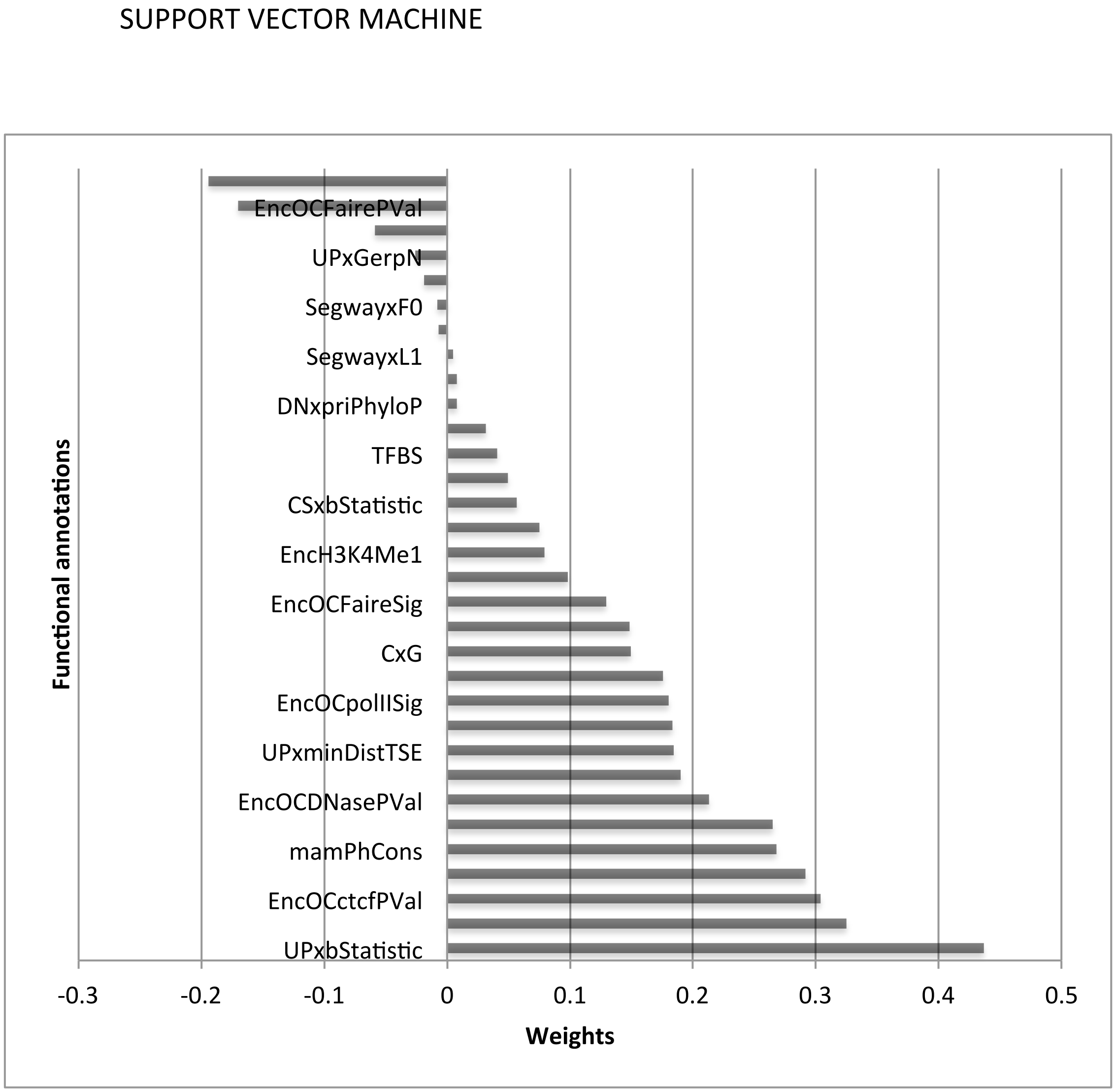

Supplementary Figure 31. Importance of annotations by Support Vector Machine

UPxGerpN	-0.0258417
TFBSPeaks	-0.0589407
EncOCFairePVal	-0.169988
UPxminDistTSS	-0.1939936

For Supplementary Tables 33-35 and Supplementary Figures 29-31:

Annotation Legend: See Kircher et al. for further details.

AltxG= interaction between Observed allele and new amino acid glycine
CSxbStatistic= interaction between canonical splice and Background selection score
CSxminDistTSS= interaction between canonical splice and Distance to closest Transcribed Sequence Start (TSS)
ConsequencexCS= canonical splice
ConsequencexI= intronic
ConsequencexUP= upstream
CxG= interaction between previous amino acid cysteine and new amino acid glycine
DNxpriPhyloP= interaction between downstream and Primate PhyloP score (excl. human)
Dst2SplTypexACCEPTOR= Closest splice site is ACCEPTOR
Dst2SplTypexDONOR= Closest splice site is DONOR
Dst2SplTypexUD= Closest splice site is undefined
Dst2Splice= Distance to splice site in 20bp; positive: exonic, negative: intronic
EncExp= Maximum ENCODE expression value
EncH3K4Me1= Maximum ENCODE H3K4 methylation level
EncH3K4Me3= Maximum ENCODE H3K4tri methylation level
EncOCCombPVal= ENCODE combined p-Value (PHRED-scale) of Faire, Dnase, polII, CTCF, Myc evidence for open chromatin
EncOCDNasePVal= p-Value (PHRED-scale) of Dnase evidence for open chromatin
EncOCFairePVal= p-Value (PHRED-scale) of Faire evidence for open chromatin
EncOCFaireSig= Peak signal for Faire evidence of open chromatin
EncOCctcfPVal= p-Value (PHRED-scale) of CTCF evidence for open chromatin
EncOCctcfSig= Peak signal for CTCF evidence of open chromatin
EncOCpolIIPVal= p-Value (PHRED-scale) of polII evidence for open chromatin
EncOCpolIISig= Peak signal for polII evidence of open chromatin
GC= Percent GC in a window of +/- 75bp
GerpN= Neutral evolution score defined by GERP++
GerpRS= Gerp element score
GerpRSpval= Gerp element p-Value
IxGerpN= interaction between intronic Neutral evolution score defined by GERP++
IxbStatistic= interaction between intronic and Background selection score
